# Infiltrating monocytes augment alternative complement activation and exacerbate inherited retinal degeneration in a mouse model

**DOI:** 10.21203/rs.3.rs-8734757/v1

**Published:** 2026-05-28

**Authors:** Wenxin Ma, Quyan Zhang, Pinghu Liu, Jingqi Lei, Carl Haugen, Lian Zhao, Robert Lee, Lijin Dong, Fusheng Tang, John Ball, Francisco M. Nadal-Nicolás, Vincent Kunze, Yue Gao, Yichao Li, Yong Zeng, Haohua Qian, Rafael Villasmil, Wan-Chi Lin, Maria Lopez-Ocasio, Steven Hockman, Pradeep Dagur, Johnny Tam, Wai T Wong, Wei Li

**Affiliations:** a Retinal Neurophysiology Section; b Genetic Engineering Core; c NEI Visual Function Core; d Flow Cytometry Core, National Eye Institute, NIH; e Flow cytometry Core, National Heart, Lung, and Blood Institute (NHLBI), NIH; f Clinical and Translational Imaging Section, NEI/NIH; g Tiresias Bio, Half Moon Bay, CA, USA.

**Keywords:** Microglia, macrophage, monocyte, Müller cell, C3, CFH, CCR2-CreER, rd10, DTA

## Abstract

In retinal degenerative disease, microglia and macrophages accumulate at sites of pathology and strongly influence disease progression, yet their distinct contributions remain unclear. To define the fate and function of infiltrating monocyte-derived macrophages (MDM) in retinal degeneration, we generated a CCR2-CreER mouse line on the *rd10* background to enable precise monocyte-specific tracking and ablation. Infiltrating monocytes rapidly downregulated CCR2 and LY6C upon entering the retina and acquired *de novo* TMEM119 and P2RY12 expression, together with a ramified, microglia-like morphology. Immunohistochemistry and transcriptomic profiling showed that a subset of these cells was cleared by resident microglia. Microglia–monocyte interactions enhanced Müller cell C3 production, whereas activated microglia increased CFB and decreased CFH expression, thereby promoting complement alternative pathway activation. Selective ablation of infiltrating monocytes reduced microglial activation and phagocytosis, suppressed Müller cell C3 expression and complement deposition, lowered proinflammatory cytokine levels, and ultimately ameliorated photoreceptor degeneration. These findings identify infiltrating monocytes as key drivers of immune dysregulation and proinflammation, highlighting them as potential targets for neuroprotective therapy.

## Introduction

Microglia are innate immune cells in the central nervous system (CNS), and their populations are heterogeneous (Masuda et al., 2020; Tan et al., 2020; Amann et al., 2023). Whether peripheral-origin macrophages constitute the composition of local microglia/macrophage homeostasis is still not fully understood (Yin et al.,2017; Amann et al., 2023). The knowledge of the population composition of local macrophages in CNS was mostly obtained from single-cell RNA sequencing (scRNA seq) data (Keren-Shaul et al., 2017; O'Korenet al.,2019; Masuda et al., 2019; Olah et al., 2021; Young et al., 2021; Marsh et al., 2022) using sorted cells with antibody labeling. The method depends on the gene expression under those conditions at that time. However, the gene expressions of microglia/macrophages, whether from local or from circulation origin, are dynamically changing during development and in the progression of pathological conditions (Yona et al., 2012; Ma et al., 2017; Chen et al., 2020). Thereby, the microglia/macrophage heterogeneity results from these non-cell fate tracing data may not be accurate (Liu et al., 2019).

Over the decades, many attempts have been made to distinguish local microglia from infiltrating macrophages. Our previous reports and other labs used a tdtomato reporter line crossed with a microglial-gene-driven CreER line such as CX3CR1-CreER (Yona et al., 2012; Ma et al., 2017), TMEM119-CreER (Baaklini et al., 2023), or LyzM-CreER (Clausen et al.,1999), for tracing the local microglia after peripheral myeloid cell turnover under tamoxifen (TAM) administration. In this transgenic strategy, the local microglia are tdtomato and Iba1 staining positive, but infiltrated peripheral macrophages are tdtomato negative and Iba1 positive in the CNS (Yona et al., 2012; Ma et al., 2017; Baaklini et al., 2023; Clausen et al.,1999). This method can clearly distinguish local microglia cells from peripheral macrophages in both the short and long term. The disadvantages are: 1) The time for peripheral myeloid cell turnover takes at least 1 month; and 2) The turnover of peripheral myeloid cells is not 100%. An alternative method for tracing peripheral macrophages is the use of a CCR2-RFP knock-in mice (Saederup et al., 2010; Dal-Secco et al., 2015). This tracing method depends on CCR2 gene expression. However, current knowledge and our previous reports showed that the CCR2 expression changes after infiltration into the retina. Therefore, it is not accurate to use CCR2-RFP knock-in mice to trace peripheral macrophages. To accurately trace peripheral macrophages, several transgenic lines have been created (Ishibashi et al., 2018; Wang et al., 2024; Paladini et al., 2025). We also generated a CCR2-CreER line and used CCR2^CreER/+^;tdT^F/+^ and CCR2^CreER/+^;tdT^F/+^;DTA^F/+^ transgenic mice for fate mapping and manipulating the infiltrated macrophages.

The function of infiltrated macrophages is another controversial area (Garré et al.,2018; Spiteri et al., 2022). Under pathologic conditions, when the blood-brain barrier (BBB) or blood-retinal barrier (BRB) is broken, the monocytes and/or monocyte progenitors in circulation infiltrate the brain (Varvel et al., 2016; Park et al., 2022; Yan et al., 2022) or the retina (Ma et al., 2017; Yu et al., 2020). These infiltrated monocytes/monocyte progenitors differentiate into macrophages within the local environment and participate in the local pathological processes. Some reports have shown that infiltrated macrophages are beneficial in aiding stroke recovery (Pedragosa et al., 2020; Park et al., 2022). However, other data indicated that infiltrated macrophages resulted in increased neuron cell death and restricted neuron cell regeneration (Varvel et al., 2016; Funatsu et al., 2022; Chen et al., 2022; Blasdel et al., 2024). To trace and explore the functions of infiltrated macrophages from the peripheral circulation, we crossed CCR2-CreER mice with tdtomato reporter with/without DTA-flox under the rd10 photoreceptor degeneration condition. We are using this triple or quadruple transgenic model not only to track infiltrated macrophages but also to study the function of infiltrated peripheral macrophages by inducing tamoxifen-mediated DTA toxin expression. Our results indicated that infiltrated macrophages can be tracked both in the short-term and long-term using CCR2^CreER/+^;tdT^F/+^ after tamoxifen induction in various pathological conditions. Infiltrated monocytes differentiate into local microglia-like cells monocyte-derived macrophage (MDM), expressing P2ry12 and TMEM119, and participate in the formation of retinal homeostasis in the later stages of the disease. Depletion of peripheral monocytes decreased peripheral macrophage infiltration and protected photoreceptor degeneration. RNA-seq data and immunostaining revealed that the ablation of CCR2-expressing cells inhibited complement C3 production in Müller cells, as well as the activation and opsonization of C3b/iC3b to photoreceptors and reduced inflammatory cytokine production in microglia.

## Results

### Generation and validation of a CCR2-CreER mouse line enabling cell-fate mapping and tracking of infiltrating monocytes

**Fig. 1.**
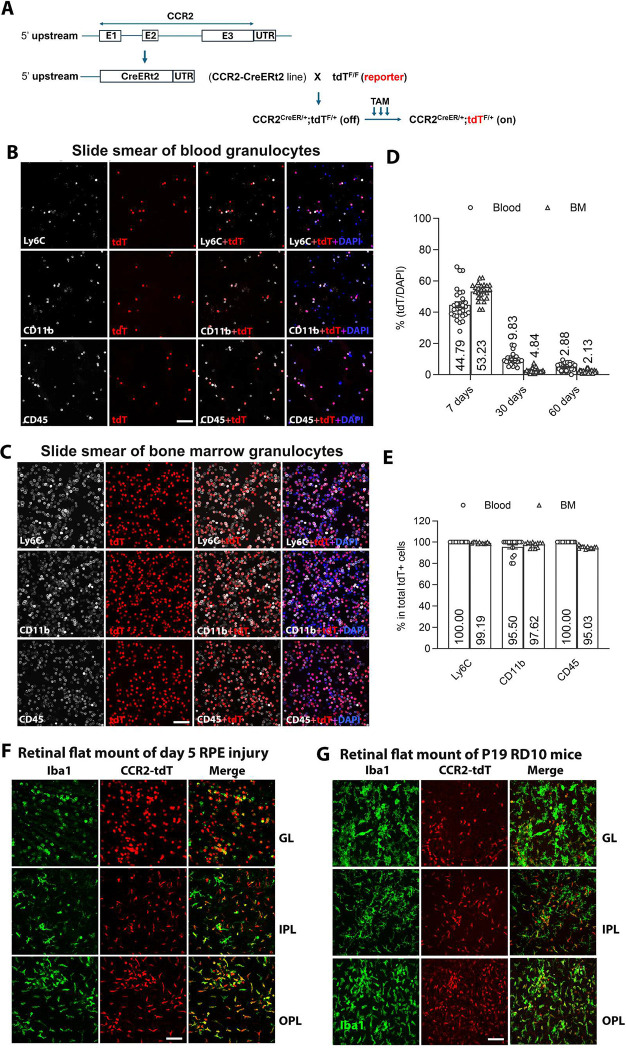
Generation and validation of CCR2-CreER knock-in transgenic line. (A) Diagram of CCR2-CreERt2 knock-in strategy. The total CCR2 coding sequence was replaced by the CreERt2 codon. CCR2-CreERt2 homozygous mice were crossed with tdT-Flox reporter mice to generate CCR2^CreER/+^;tdT^F/+^ mice, the tdtomato (tdT) signal will be turned on after tamoxifen induction. (B) & (C) The images showed granulocytes in the blood (B) and bone marrow (C), respectively, after 1-week of tamoxifen induction. The cells were stained with Ly6C, CD11b and CD45. The scale bar = 60 μm. (D) Histogram showing the percentage of tdT+ cells among total granulocytes; the percentage decreased further after 30 and 60 days of tamoxifen induction. (E) Percentage of CD11b, CD45 and Ly6C expression in the total tdT+ cells in the blood and bone marrow after 1 week of tamoxifen induction. (F) Retinal flat mount images showed tdT+ cells infiltration into the retinal ganglion cell layer (GL), inner plexiform layer (IPL), and outer plexiform layer (OPL) in CCR2^CreER/+^;tdT^F/+^ mice retina at day 5 of RPE cell injury induced by NaIO3. Tamoxifen was administered 1 week before NaIO3 administration. Scale bar = 60 μm. (G) Retinal flat-mount images showed tdT+ cell infiltration into the retinal GL, IPL, and OPL at P19 of rd10;CCR2^CreER/+^;tdT^F/+^ mice. Tamoxifen was administered at p14, p15, and p16. The scale bar = 60 μm.

To track the fate of peripheral monocytes infiltrating the degenerating retina, we generated a CCR2-CreER mouse line by replacing both copies of the CCR2 coding sequence with a sequence coding for CreERT2, a tamoxifen-inducible Cre recombinase protein (Saederup et al., 2010; Fig. 1A). These mice were then crossed to a tdT-flox line (Madisen et al. 2010) to generate heterozygous CCr2^CreER/+^;tdT^F/+^ mice. In the absence of tamoxifen (TAM) induction, these animals did not show detectable tdT expression in circulating or bone marrow monocytes (Fig. 1A, Fig. 1 suppl. A–C), indicating the absence of uninduced Cre recombinase activity (“leakiness”) in this line. Upon induction with tamoxifen administration, tdT expression was detected in 44.79% and 53.23% of granulocytes in blood and bone marrow, respectively at 1-week post-induction (Fig. 1. B, C, D). As the turnover of monocytic cells in the blood and bone marrow is important in identifying resident, long-lived microglia in the CX3CR1^CreER/+^;tdT^F/+^ model (Yona et al., 2012; Ma et al., 2017), we assessed the extent of tdT+ cell turnover in blood and bone marrow at 1 month and 2 months post-tamoxifen induction. We found that the proportions of tdT+ cells decreased to 9.83% and 4.84% in the blood and bone marrow, respectively, at 1 month, and then to 2.88% and 2.13% at 2 months (Fig. 1D). A small proportion of tdT+ cells can’t turn over even after 2 months. The tdtomato+ cells are also expressed Ly6C, CD11b and CD45 (Fig. 1E). In the tamoxifen-induced healthy young 2-month-old adult CCR2^CreER/+^;tdT^F/+^ mice without retinal degeneration, tdT+ cells were absent in the healthy retina (Fig. 1. Suppl. D). In contrast, numerous tdT+ cells were observed in retinas in models of retinal degeneration, including the sodium iodate(NaIO3) -induced retinal pigment epithelial (RPE) cell injury model (Fig. 1F), the rd10 photoreceptor degeneration model (Fig. 1G), and the light-induced photoreceptor degeneration model (Fig. 1, Suppl. E, F). These results demonstrate that the CCR2-CreERt2 mouse line is a suitable model for tracking peripheral monocytes that infiltrate the retina in models of retinal degeneration.

### Monocyte-derived macrophages (MDM) express CCR2 and Ly6C transiently in the retina but can be tracked by CCR2^CreER/+^;tdT^F/+^ mouse line

**Fig. 2.**
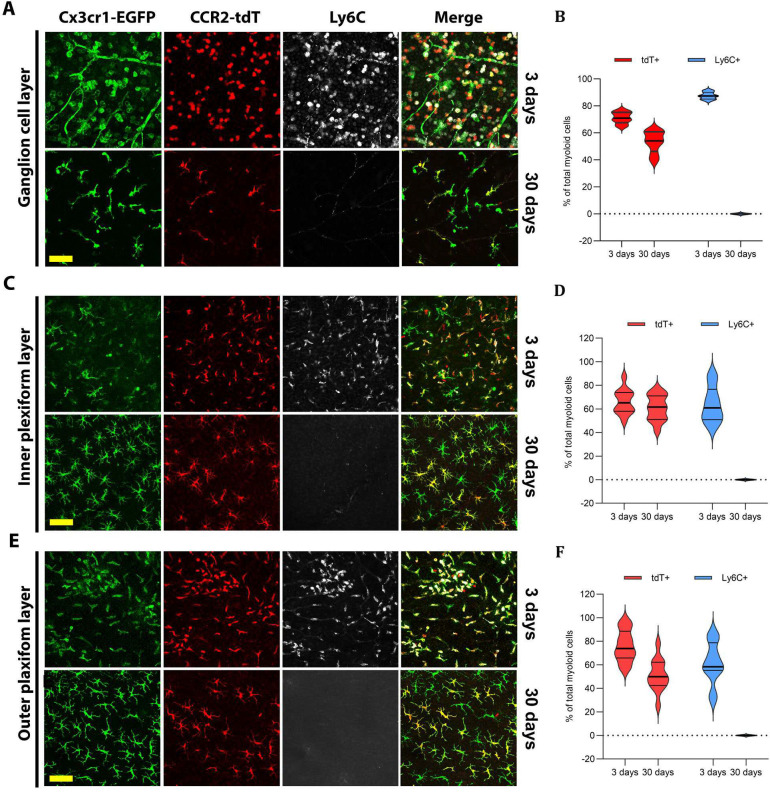
The CCR2^CreER/+^;tdT^F/+^ mouse line can be used to trace peripheral macrophages for the long term. Infiltrated CCR2-tdT+ cells can be seen in CCR2^CreER/+^;tdT^F/+^;CX3CR1^EGFP/+^ mice retina, not only at an early stage of 3 days (upper panel) but also at a later stage at 30 days (lower panel) in GL(A), IPL(C), and OPL(E) post-RPE injury with NaIO3 administration. However, infiltrated CCR2-tdT+ cells expressing Ly6C only be detected at the early stage of 3 days of injury, but were terminated at a later stage of 30 days (A-F). Scale bar=60 μm. (B), (D), and (F) show the percentage of CCR2-tdT+ and Ly6C+ cells in all myeloid cells in the retinal GL, IPL, and OPL, respectively. At 30 days after RPE injury, no Ly6C+ cells are detected (0%), but tdT+ cells remain in the retina.

Previous studies have shown that macrophages normally resident in tissue are joined by CCR2+, LY6C+ monocytes recruited from the systemic circulation under inflammatory conditions; however, these recruited cells were initially thought to be cleared from the retina as immunopositivity for CCR2 and Ly6C cell markers declined with time (Ajami et al., 2011; Leuschner et al., 2012). To further understand the fate of infiltrated monocytes in the context of retinal degeneration, we used the model of NaIO_3_-induced retinal pigment epithelium (RPE) injury in CX3CR1^EGFP/+^;CCR2^RFP/+^ (Dal-Secco et al., 2015) double transgenic mice to trace the fate of retinal microglia (which are marked with EGFP only) vs. infiltrated monocytes (which are marked with both EGFP and RFP). The results indicated that RFP+ monocytes infiltrated into the ganglion cell layer (GL) (Fig. 2. Suppl. A), inner plexiform layer (IPL) (Fig. 2. Suppl. C), and outer plexiform layer (OPL) (Fig. 2. Suppl. E) 3 days after RPE injury, but the number of RFP+ cells decreased after 7 days and was undetectable by 30 days after NaIO_3_ administration (Fig. 2. Suppl. B, D, F). These observations, however, do not distinguish between the possibilities of monocyte disappearance vs. the downregulation of CCR2 expression in persisting monocytes. Similar experiments performed on CCR2^CreER/+^;tdT^F/+^;CX3CR1^EGFP/+^(CCR2^CreER^-CX3CR1) mice following tamoxifen induction demonstrated that marked tdT+ monocytes were present in the GL (Fig.2. A), IPL (Fig. 2. C), and OPL (Fig. 2. E) at 3 days and were also persistent in the retina at 30 days after NaIO_3_ administration (Fig. 2. B, D, F). We further confirmed using another monocyte marker, Ly6C (Miyake et al., 2024), which is typically absent in the healthy retina but appears after RPE cell injury. We observed that tdT+ monocytes were Ly6C+ 3 days post-injury but became Ly6C-negative after 30 days (Fig. 2. A–F). Taken together, these findings indicate that both monocytic markers of CCR2 and Ly6C were expressed only transiently in infiltrated monocytes (<7 days) and did not enable longer-term tracking of infiltrated monocytes, in contrast to the persistence of tdT-labeling in infiltrating monocytes in the CCR2^CreER/+^;tdT^F/+^ mouse model that can support long-term tracking of infiltrated monocytes.

### Infiltrated monocytes differentiate to resemble resident microglia with respect to morphology and marker identity with time

**Fig. 3.**
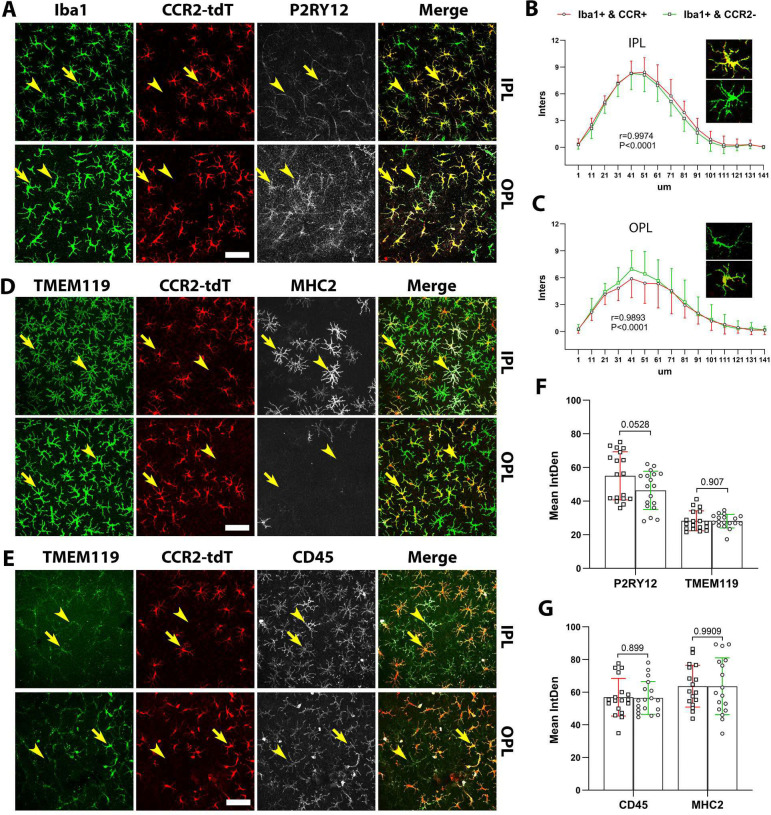
Infiltrated monocytes differentiated into microglia-like cells (MDM) in the mouse retina and rebuilt a new homeostasis with local microglia cells. (A) Retinal flat mount images showed infiltrated CCR2-tdT+ macrophages in IPL (upper panel) and OPL (lower panel) expressing Iba1 and P2ry12 after NaIO3-induced RPE injury 40 days. Arrowheads indicated Iba1+ & CCR2-tdT− resident microglia, arrows indicated Iba1+ & CCR2+ infiltrated MDMs. Scale bar=60 μm. (B) & (C) The morphology analysis showed no differences in IPL (B) and OPL (C) between resident Iba1+ & CCR2− microglia and infiltrated Iba1+ & CCR2+ macrophages (MDM) using Sholl analysis (Image J). (D) & (E) Retinal flat mount images showed that both infiltrated MDMs and local microglia also expressed TMEM119, MHC2 and CD45 in IPL (upper panel) and OPL (lower panel) after NaIO3 caused RPE injury 40 days. Arrowheads indicated Iba1+ & CCR2-tdT− resident microglia, arrows indicated both Iba1+ & CCR2+ infiltrated MDM. Scale bar=60 μm. (F) The mean-intensity analysis showed that P2ry12 and TMEM119 expression are not different between resident microglia and infiltrated MDMs. (G) The mean intensity analysis showed that CD45 and MHC2 expression was not significantly different between resident Iba1+ & CCR2− microglia and infiltrated Iba1+ & CCR2+ MDMs.

Having established that infiltrated monocyte-derived macrophages (MDM) are persistent in the retina over the long term, we used the CCR2^CreER/+^;tdT^F/+^ mouse line and NaIO_3_ injury model to examine their longer-term fate post-infiltration. At 40 days post- injury, following the acute phase of the injury and the re-establishment of stable myeloid cell numbers (Ma et al., 2017; Chen et al., 2020, 2022), immunohistochemical analyses in flat-mounted retina showed that CCR2-tdT+ MDMs were found juxtaposed alongside CCR-tdT− resident microglia in a single contiguous mosaic pattern in the IPL and also in the OPL (Fig. 3A). Both CCR2-tdT+ and CCR2-tdT− cell populations were immunopositive for IBA1 as well as for P2RY12, a marker attributed to ramified, “resting” microglia. Both populations showed similar ramified morphologies in their processes that were not significantly different on Sholl analysis in both the IPL and OPL (Fig. 3B, C, Fig. 3. Suppl 1).

Immunohistochemical analysis demonstrated that both cell populations were immunopositive for P2RY12 and TMEM119 (Fig. 3A, D), with similar levels of immunopositivity (Fig. 3F). These results indicated that the tdT+ MDMs differentiated to resemble resident microglia and are incorporated into a single spatial mosaic of ramified cells, suggestive of integration into a common steady-state for myeloid cell homeostasis in the retina. The ability of infiltrated monocytes to acquire immunopositivity for markers traditionally associated with microglia (P2RY12 and TMEM119) indicate that the definition of distinct myeloid cell populations on single-cell RNAseq or flow-cytometry analyses based on these markers may potentially confound cells derived separately from infiltrated monocytes and resident microglia. Other markers for distinguishing resident microglia and MDMs, such as MHC2 and CD45, also showed positive staining in both cell types, with similar expression levels (Fig. 3. D, E, G). We also found the time-dependent acquisition of common markers by MDMs in other models of retinal degeneration, including the light-induced retinal injury model (Fig. 3. Suppl. 2 and 3) and the rd10 model for inherited retinal degeneration (Fig. 3. Suppl. 4).

### Protection of retinal function after ablation of CCR2-expressing cells in rd10 mice

**Fig. 4.**
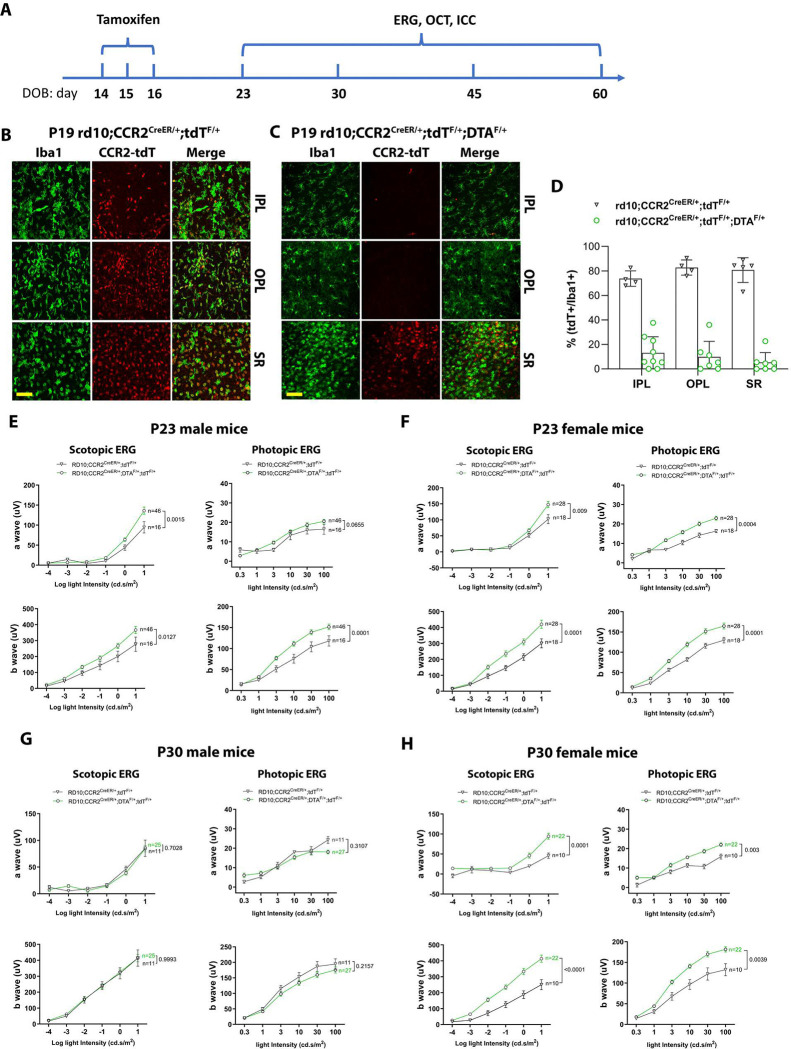
Ablation of peripheral monocytes protects retina function in the rd10 mice of the retinal degeneration model. (A) Diagram shows the tamoxifen administration and the ERG, OCT and immunocytochemistry (ICC) detection time point. (B) & (C) Retinal flat mount images showed infiltrated CCR2-tdT+ macrophages in IPL (the top panel), OPL (the middle panel), and SR (the bottom panel) in P19 rd10;CCR2^CreER/+^;tdT^F/+^ and rd10;CCR2^CreER/+^;tdT^F/+^;DTA^F/+^ mice after tamoxifen administration. Scale bar=100um. (D) The percentage of tdT+ cell in all Iba1+ and tdT+ cells. (E) The ERG results from P23 male rd10 mice. F. The ERG results from P23 female rd10 mice. G. The ERG results from P30 male rd10 mice. H. The ERG results from P30 female rd10 mice. All data were analyzed with two-way ANOVA.

The functions of infiltrated peripheral macrophages have been extensively studied (Varvel et al., 2016; Pedragosa et al., 2020; Park et al., 2022; Funatsu et al., 2022; Chen et al., 2022; Blasdel et al., 2024), but whether they are beneficial or detrimental remains unclear. We crossed our CCR2^CreER/CreER^;tdT^F/F^ mouse line with rd10 and DTA-flox mice to generate rd10;CCR2^CreER/+^;tdT^F/+^ (rd10-CCR2) and rd10;CCR2^CreER/+^;tdT^F/+^;DTA^F/+^ (rd10-CCR2-DTA) mice to explore photoreceptor degeneration with and without depletion of CCR2-expressing monocytes. The rd10 mice have a spontaneous missense point mutation in *PDE6B* (cGMP phosphodiesterase 6B, rod receptor, beta polypeptide) and have been used as a Retinitis pigmentosa (RP) model. In rd10 mice, rods begin to degenerate around P16, with maximum cell death occurring between P21 and P25 in a central to peripheral gradient. By P60, rods are no longer detectable and only a few cones remain (Pennesi et al., 2012). Following the photoreceptor cell death timeline, we administer tamoxifen from P14 to P16 to both groups of mice and measure retinal function with Electroretinography (ERG) at P23, P30, P45, and P60 (Fig. 4A). Before initiating the experiments, we tested the efficacy of CCR2+ cell depletion. The CCR2^CreER/+^;tdT^F/+^;DTA^F/+^ and CCR2^CreER/+^;tdT^F/+^ mice were administered with tamoxifen (TAM) for 3 days consecutively through IP injection, and then the blood and bone marrow granular cells were collected on the 4^th^ day. The results demonstrated that 97.8% and 96.8% of tdT+ cells were depleted in the blood and bone marrow, respectively (Fig. 4, Suppl. 1A–E), indicating that the depletion was quite efficient. The retinas from both rd10-CCR2 and rd10-CCR2-DTA groups after tamoxifen administration were harvested at P19 and stained with Iba1 (Fig. 4 B, C). The results revealed that tdT+ cells are much decreased in rd10-CCR2-DTA mice (Fig. 4D). The ERG results demonstrated that the b wave amplitude in rd10-CCR2-DTA mice is significantly higher than in rd10-CCR2 mice at p23 in both scotopic and photopic condition in both male (Fig. 4E) and female (Fig. 4F) mice. These results indicate that the depletion of CCR2-expressing monocytes protects not only photoreceptors but also other retinal cells, like bipolar and Müller cells. In P23 female rd10-CCR2-DTA mice, the amplitude of a wave also is higher significantly in both scotopic and photopic conditions indicating that depletion of monocytes protected or delayed rod degeneration and cone mediated responses (Fig. 4F). This protection continues to P30 female rd10-CCR2-DTA (Fig. 4H) but declined at P45 (Fig. 4. Suppl. 2. A & B) and P60 (Fig. 4. Suppl. 2. C & D) even the amplitudes of b wave trend higher in rd10-CCR2-DTA. Taken together, the rd10-CCR2-DTA mice can be used to manipulate CCR2+ monocyte infiltration. Depletion of CCR2+ cells in the systemic circulation can decrease their infiltration into the retina and protect retinal function in rd10 mice of the RP model.

### Protection of retinal structure after ablation of CCR2 expression cells in the rd10 and RPE injury model

**Fig. 5.**
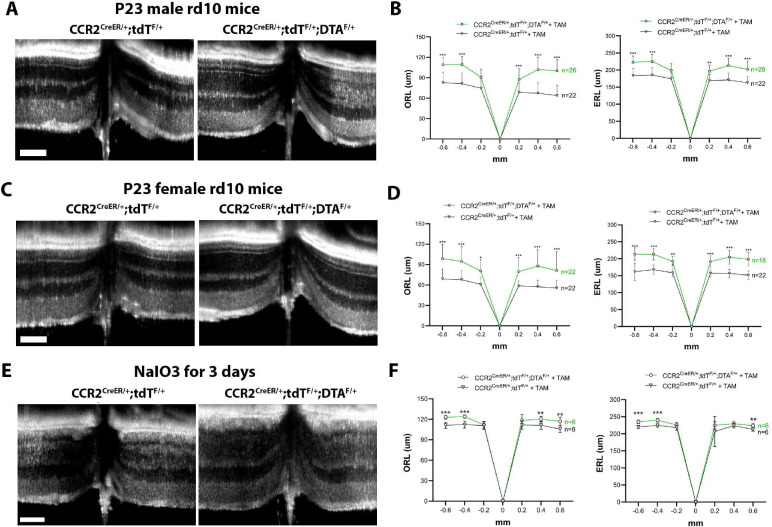
Depletion of peripheral monocytes preserves the retinal thickness in mouse retinal degeneration. (A) The representative Bioptigen domain Optical Coherence Tomography (OCT) images showed retinal layers from superior to inferior in P23 rd10;CCR2^CreER/+^;tdT^F/+^ (Left) and rd10;CCR2^CreER/+^;tdT^F/+^;DTA^F/+^ (Right) male mice under tamoxifen induction. Scale bar = 0.1mm. (B) The thickness of the outer retinal layer (ORL, left) and the entire retinal layer (ERL, right) in P23 male mice. (C) The OCT images showed retinal thickness in P23 female rd10 mice. Scale bar = 0.1 mm. (D) The results of retinal thickness measurement. The ORL (Left) and ERL (Right) thickness in P23 female mice retinas. (E) RPE injury induced by NaIO3 causes retinal degeneration. The images showed representative retinal OCT images obtained 3 days after RPE injury. Scale bar = 0.1mm. (F) The results of retinal thickness measurement of the ORL (Left) and ERL (Right). All data were analyzed with an unpaired two-tailed Student’s t-test or with a Mann-Whitney test. * p<0.05, **P<0.01, *** P<0.001.

Ablation of CCR2-expressing cells in rd10 mice protects retinal function, as indicated by ERG results. To examine structure, we measured the thickness of the retina in rd10-CCR2 and rd10-CCR2-DTA groups using spectral domain optical coherence tomography (SD-OCT) (Knott et al., 2010; Pennesi et al., 2012). To measure the thickness of the outer retina layer (ORL) and entire retinal layer (ERL) more easily and precisely, the ORL was measured from RPE basal to OPL and included OPL; the ERL was measured from RPE basal to ganglion cell layer/retinal nerve fiber (GL/RNF) (Fig. 5. Suppl. 1). We measured 6 positions: 0.6mm, 0.4mm, and 0.2mm superior to optical nerve head (ONH) and 0.2mm, 0.4mm, and 0.6mm inferior to the ONH. In OCT images from P23 mice, we can see the retinal thickness in rd10-CCR2-DTA mice is much thicker than rd10-CCR2 mice (Fig. 5. A & B: male; C & D: female) with both ORL and ERL. Surprisingly, similar results were observed in some areas of P30 (Fig. 5. Suppl. 2A–B), P45 (Fig. Suppl. 2C–D), and P60 mice (Fig. Suppl. 2E–F), despite continued degeneration. In P30 male and female mice, the results showed a large variation, but the trend was consistent. These results indicated that depletion of CCR2+ cells not only protects the retinal function but also preserves or delays retinal structure degeneration. This protection seems to extend not only to photoreceptors but also to other retinal layers. The results also show that tamoxifen has a protective effect against retinal degeneration, as reported in our previous work (Wang et al., 2017; data not shown).

In the RPE injury model induced by NaIO3, we measured retinal thickness using OCT at 1, 3, and 8 days after NaIO3 administration. The retinal thickness is not significantly different between with and without CCR2 positive cell depletion at day 1 RPE injury (data not shown). However, at 3 days (Fig. 5E) and 8 days (Fig. 5. Suppl. 3A) post RPE injury, the retinal thickness of ONL and ERL after depletion of peripheral monocytes in mice is significantly higher than in non-depletion mice (Fig. 5F, Fig.5. Suppl. 3B). Taken together, these results indicate that blocking peripheral monocyte infiltration protects against retinal injury in a disease-agnostic manner.

### Ablation of CCR2-expressing monocytes decreases microglia clustering and phagocytosis capability in rd10 mice retina

**Fig. 6.**
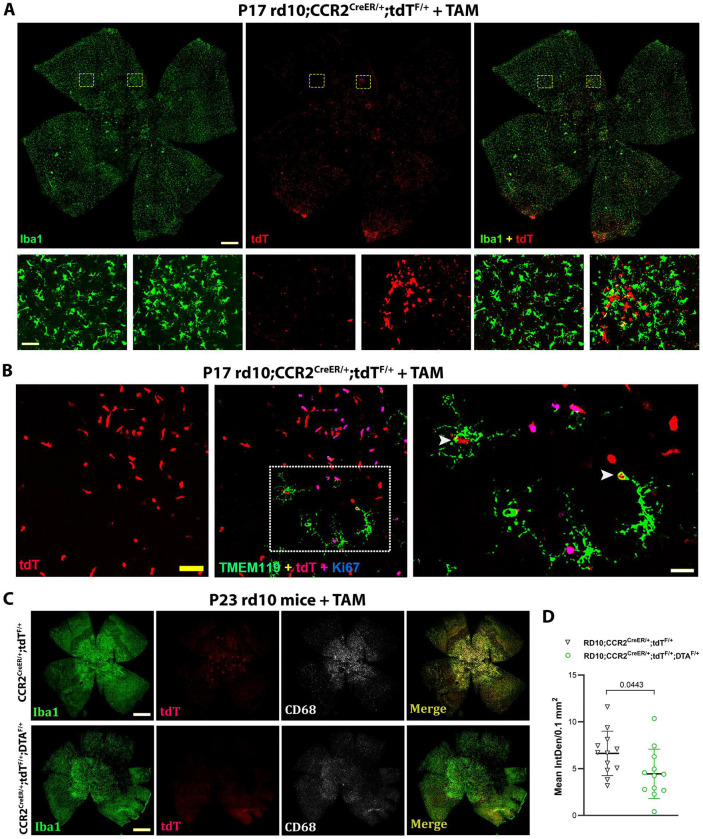
Ablation of peripheral monocytes decreases the microglia cell clustering and the ability of phagocytosis. (A) tdT+ peripheral monocytes caused microglia clustering (whole mount, p17) in rd10;CCR2^CreER/+^;tdT^F/+^ mice retina. Scale bar = 1.2 mm. The bottom images showed magnified views of the selected areas. The distribution of microglia is more even in the area without MDMs, but in the CCR2+ cells area, microglia lose this even distribution and form clusters. the Scale bar=160 μm. (B) Infiltrated CCR2+ cells continue to divide, as indicated by Ki67 staining, around resident microglia, as TMEM119+ cells. Scale bar = 124 μm. The enlarged image and arrowhead showed resident microglia phagocytosing CCR2+ cells. Scale bar = 53μm. (C) Whole retinal flat mount imaging of the P23 mouse retina showed CD68 expression. Scale bar=1.2 mm. (D)The mean intensity of CD68 expression from Figure C.

There are several methods of decreasing/blocking monocyte infiltration, such as the knockout of the CCR2 receptor in mice (Ma et al., 2017; Tian et al.,2017; Tian et al., 2022) or using a CCR2 receptor antagonist (Morganti et al., 2015; Alemán-Ruiz et al., 2023; Jin et al., 2024). Both methods can decrease monocyte infiltration and local inflammation. The depletion of CCR2+ cells in the circulation, both before and at the onset of disease development, significantly decreased monocyte infiltration (Fig. 4C, D). Here, we assessed the levels of activation and phagocytosis in resident microglia with and without CCR2+ cell depletion. At P17 rd10 mouse retina, we found that the microglia formed clusters around the infiltrated CCR2+ cells (Fig.6A), indicating that infiltrated monocytes attracted and activated local microglia, causing dyshomeostasis. These microglia clusters and dyshomeostasis were significantly decreased after CCR2+ cell depletion (Fig. 6. Suppl. A). We found that resident microglia recognized infiltrated monocytes as alien cells and attempted to remove them through phagocytosis (Fig. 6B, indicated by arrowhead; Fig. 6 Suppl. B, arrow indicated). These rejection responses are reminiscent of the process used to eliminate xenobiotics. The phagocytosis ability, as indicated by CD68 staining, was also significantly decreased in the retina of CCR2+ cell-depleted mice (Fig. 6C, D). The battle between resident microglia and infiltrated monocytes exacerbates local dyshomeostasis and damages photoreceptors. The fluorescein isothiocyanate (FITC)-conjugated peanut agglutinin (PNA) staining showed that the PNA-occupied area is significantly increased in the absence of monocyte infiltration (6. Suppl. C, D). These results demonstrate that infiltrated monocytes promote microglia activation and local dyshomeostasis, and exacerbate photoreceptor cell death.

### Infiltrated CCR2-expressing monocytes activate the alternative complement pathway and increase the detrimental conditions in the rd10 mouse retina

**Fig. 7.**
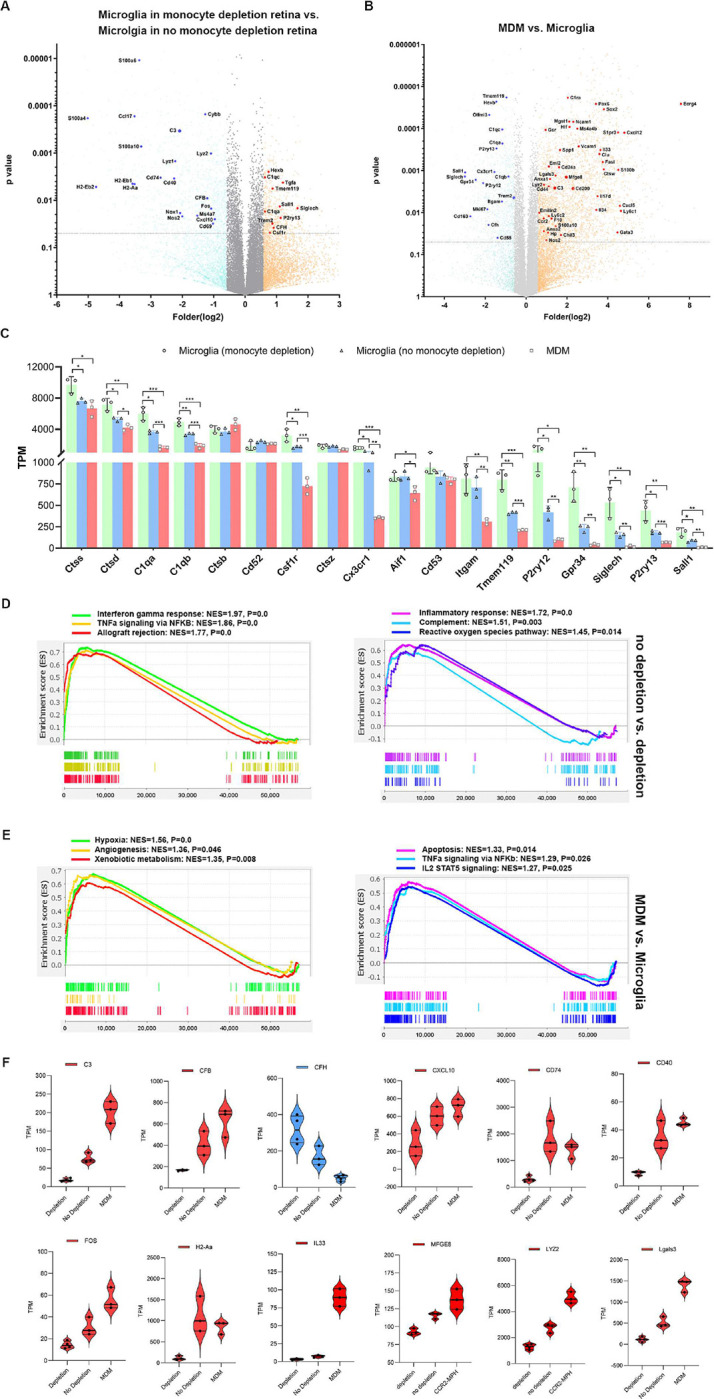
Bulk RNA Sequencing results showed that CCR2+ MDM infiltration promoted the resident microglia activation and a distinct function from resident microglia. (A) The volcano plot showed differential gene expression between microglia with and without CCR2+ cell depletion in P23 rd10 mice. (B) The volcano plot revealed distinct gene expression patterns in MDM versus non-depleted retinal microglia in P23 rd10 mice. (C) The microglial signature genes in microglia with CCR2+ cell depletion, microglia without CCR2+ depletion, and MDM in the P23 rd10 mouse retina. (D) GSEA analysis compared microglia between the no CCR2+ cell-depletion vs. the CCR2+ cell-depletion conditions. (E) GSEA analysis showed the comparison results of MDM vs. resident microglia in P23 rd10-CCR2 mice. (F) Some representative genes in the GSEA results were shown in a violin plot.

To understand the functions of resident microglia and the effect of MDMs in the retinal degeneration of rd10 mouse retina, we carried out bulk RNA seq on the FACS-sorted resident microglia and MDMs. The resident microglia were sorted with CD11b+ & tdT− cells and the MDMs were sorted with tdT+ cells. We compared different gene expression (DGE) of the microglia in depletion (CCR2^CreER/+^;tdT^F/+^;DTA^F/+^ rd10 mice) versus non-depletion (CCR2^CreER/+^;tdT^F/+^ rd10 mice), we found that resident microglia without MDM expressed significantly higher of microglia signature genes, like *TMEM119, P2RY13, HEXB, C1QA, CSF1R, TREM2*, etc. (Fig. 7A, C), but decreased inflammation and interferon gamma response genes, such as *CD74, CXCL10, CD40, CCL17, NOS2, H2-Aa*; complement factors *C3* and *CFB*; hypoxia involved genes *FOS, S100a4*, and phagocytosis genes *LYZ1, LYZ2, CYBB*, etc. (Fig. 7A, F; Fig. 7. Suppl.). The surprising gene is *TREM2*, whose expression is higher in microglia without MDM infiltration. The DGE analysis from MDM, comparing resident microglia in CCR2^CreER/+^;tdT^F/+^ rd10 mice retinas, elucidated opposite results (Fig. 7B, C). Infiltrated MDM expressed microglia signature genes but significantly less than resident microglia. MDM expressed significantly higher macrophage genes, such as *F10, Emilin2, C3, Anxa2, Chil3, Mgst1*, and *S100A10* (Haage et al., 2019), as well as monocyte genes *CCR2, Ly6C1, and Ly6C2*. Infiltrated MDMs expressed significantly higher levels of subretinal macrophage genes (O’Koren et al., 2019), such as *SPP1, LYZ2, Lgals3* (Fig. 7B, F; Fig. 7. Suppl.). Meanwhile, MDM expressed other function genes, such as progenitor genes PAX6 and SOX2, cell-adhesion genes VCAM1, NCAM1, and CD44, chemokines and cytokines such as CXCL12 and CXCL5, and IL33, etc.

Gene enrichment analysis indicated that the function of microglia with CCR2+ cell infiltration is involved in the interferon response, TNF-α-NFKb signaling, inflammation response, complement activation, and Reactive Oxygen Species (ROS) production, among others (Fig. 7D; Suppl. Table 1). The gene enrichment analysis of MDMs demonstrated that MDMs were involved in estrogen response, hypoxia, xenobiotic metabolism, apoptosis, and TNF-α-NFKb signaling (Fig. 7E; Suppl. Table 2). The xenobiotic metabolism-enriched genes could be involved in the phenotype of the resident microglia phagocyte MDMs (Fig. 6B, Fig. 6 Suppl. B). We determined CD74 and MHC2 expression, which are among the most common genes in the inflammation and interferon response signaling pathways (Fig. 7. Suppl. 2). The results demonstrated that CD74 and MHC2 production were significantly decreased in subretinal microglia in MDM-absent retina (Fig. 7. Suppl. 2A–D). Taken together, the gene profile data and immunostaining results demonstrated that, in the rd10 retina, microglial function without CCR2+ monocyte infiltration is closer to homeostasis, characterized by increased microglia signature genes and decreased inflammatory conditions.

**Fig. 8.**
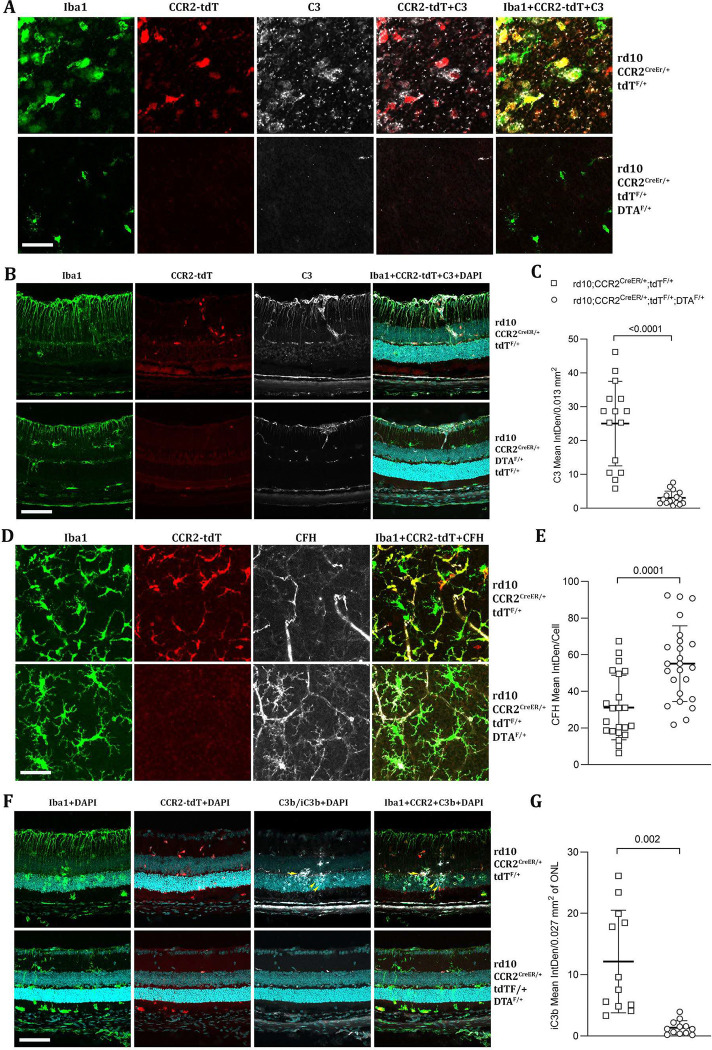
Ablation of peripheral macrophages decreased C3 production and C3b/iC3b deposition. (A) Iba1 (green) and C3 (white) staining in a flat mount of retinal outer nuclear layer (ONL) of P19 rd10;CCR2^CreER/+^;tdT^F/+^ and rd10;CCR2^CreER/+^;tdT^F/+^;DTA^F/+^ mice after tamoxifen induction. MDM were labeled with tdTomato (red). C3 (white) is expressed not only in microglia and MDMs, but also in Müller cells in the retina of RD10;CCR2^CreER/+^;tdT^F/+^ ; the C3 staining was significantly decreased in the retina after CCR2+ cell depletion (rd10;CCR2^CreER/+^;tdT^F/+^;DTA^F/+^ mice). Scale bar = 30 μm. (B) Iba1 (green) and C3 (white) staining in retinal sections of P23 retinas confirmed that C3 was significantly expressed in Müller cells and deposited on the photoreceptors in the retina of rd10;CCR2^CreER/+^;tdT^F/+^ , but decreased in the retina of rd10;CCR2^CreER/+^;tdT^F/+^;DTA^F/+^ mice. Scale bar = 60 μm. (C) The mean intensity of C3 expression in ONL (data A). (D) Iba1 (green) and CFH (white) staining on retinal flat mount of P23 rd10;CCR2^CreER/+^;tdT^F/+^ and rd10;CCR2^CreER/+^;tdT^F/+^;DTA^F/+^ mice. CFH expression decreased significantly in microglia in rd10;CCR2^CreER/+^;tdT^F/+^ mice. Scale bar = 30 μm. (E) The quantification of the mean intensity of CFH expression of each microglia. (F) Retinal microglia promote the conversion of C3 to C3b/iC3b under conditions of CCR2+ cell infiltration (rd10;CCR2^CreER/+^;tdT^F/+^), and increase the deposition of converted C3b/iC3b on photoreceptors. Arrow showed C3b/iC3b in microglia, arrowheads showed the C3b/iC3b deposition on photoreceptors. Scale bar = 60 μm. (G) The quantification of the mean intensity of C3b/iC3b in ONL of the retina.

**Fig. 9.**
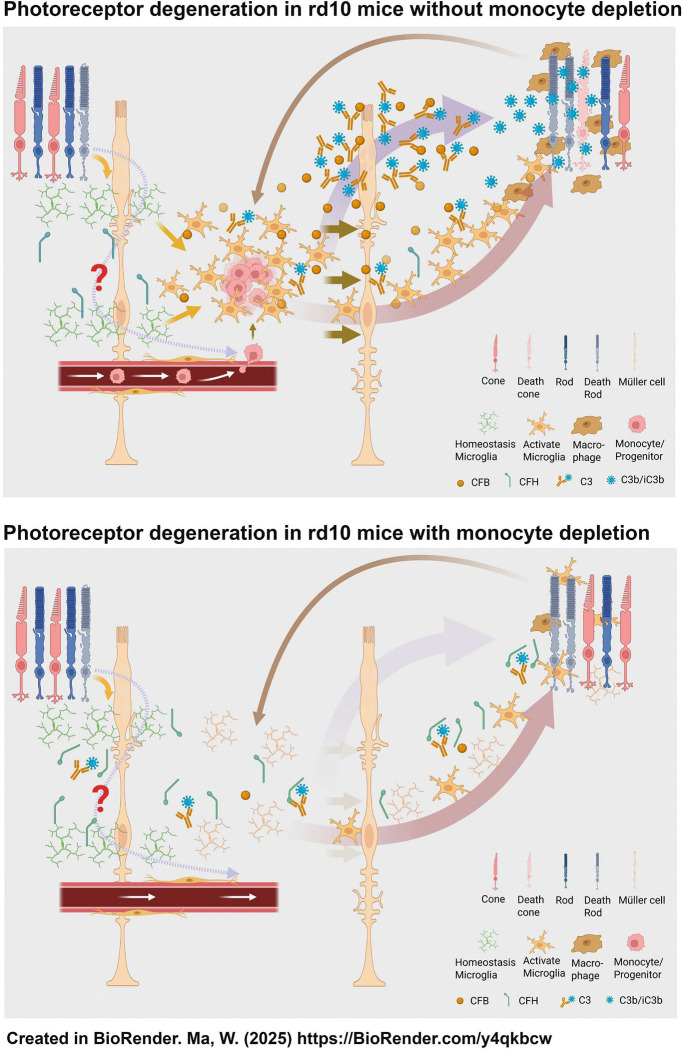
A diagram of the photoreceptor death in the retina of rd10 mice. In rd10 mice, a mutation in Pde6b initially leads to rod cell death, which activates microglia and Muller cells, causing local dyshomeostasis through complex factors we do not fully understand. These results disrupted the Retina-Blood Barrier (BRB) and released chemokines that attracted circulating CCR2+ monocytes/monocyte progenitors into the retina. CCR2+ monocytes/monocyte progenitors are believed to be “alien” cells by local immune cells, microglia, and attract microglia to form the clusters. Microglia tried to clear these alien cells through phagocytosis. The interaction of microglia and monocytes further stimulates Müller cells to produce C3. In parallel, the activated microglia increased CFB production and decreased CFH expression. The results induced a significant conversion of C3 to C3b/iC3b, which subsequently deposited on the photoreceptor, leading to increased photoreceptor death. In the retina without CCR2+ cell infiltration, there was no interaction between CCR2+ monocyte/monocyte progenitor cells and microglia. Microglia maintained a more homeostatic state, continued producing CFH, and inhibited CFB production. Müller cells also decreased or ceased production of C3, resulting in reduced C3b/iC3b conversion and deposition and reduced photoreceptor death.

The gene profile indicated that the alternative complement pathway is the primary response in rd10 retinal degeneration, characterized by increased *C3* and *CFB* expression, but decreased *CFH* expression (Fig. 7A, B, F). The immunohistochemistry staining of C3 showed that C3 was expressed not only in microglia and MDMs, but also strongly expressed in Müller cells, and had a significantly higher expression in the retina with MDM infiltration (Fig. 8A–C; Fig. 8. Suppl. 1 & 2). However, the CFH staining showed the opposite result, with significantly higher expression in retinal microglia without MDM infiltration (Fig. 8D, E). These results indicated that microglia could regulate C3 activation and deposition by producing CFH. We stained iC3b/C3b on the retinal section (Fig. 8F). The results showed that iC3b/C3b was expressed/deposited in microglia and photoreceptor cells with MDM infiltration but significantly decreased in CCR2+ cell-depleted retina (Fig. 8F, G). This could be due to the decreased CFH expression and increased CFB expression (Fig. 7F) in microglia with MDM infiltration. In summary, CCR2+ monocyte infiltration in the rd10 mouse retina promotes local inflammation, and more importantly, it activates the alternative complement pathway by increasing C3 and CFB, while inhibiting CFH production. This leads to increased iC3b/C3b deposition on the photoreceptor, accelerating the photoreceptor cell death (Fig. 9. Sweigard et al., 2015).

## Discussion

Our results indicated that infiltrated CCR2+ cells transiently express CCR2 and LY6C (<7 days) in the retina and later develop into TMEM119 and P2ry12-positive microglia-like cells in the later stage of NaIO3-induced RPE damage model. These microglia-like monocyte-derived macrophages (MDM) contribute to local macrophage homeostasis and coexist with local microglia (we traced them for more than 1 year). We also found that resident microglia express CD45^high^ and MHC2+ during inflammation. Therefore, the clustering-based classification of retinal macrophages under pathological conditions, based on gene expression profiles from single RNA-seq, is not accurate. For accurate clustering of retinal macrophages under pathological conditions, it is necessary to sort resident microglia and MDMs separately for single-cell RNA sequencing.

Photoreceptor death triggers the release of signals, a crucial aspect of the body's response to damage that plays a key role in inflammation and tissue repair. These signals include lipids, such as lysophosphatidylcholine (LPC) and fractalkine (CX3CL1), as well as nucleotides like ATP and UTP. These signals not only activate resident microglia but also activate recruited circulating monocytes. In the photoreceptor degeneration rd10 model, photoreceptor death leads to monocyte infiltration, accompanied by blood-retina barrier (BRB) leakage, which attracts local microglia that attempt to clean up and maintain local homeostasis. We found that the interaction of microglia and monocytes, or the phagocytosis of monocytes, directly or indirectly stimulates Müller cells to release complement C3. The level of CFH in microglia was inhibited, but CFB expression was increased. This ultimately led to the activation of the alternative complement pathway, promoting the conversion of C3 to C3b/iC3b, which deposited on the photoreceptor and accelerated cell death (Fig. 9). Monocyte infiltration was blocked by CCR2+ cell depletion before the onset of photoreceptor degeneration, resulting in significantly reduced C3 production and C3b/iC3b deposition by inverted C3, CFB and CFH production (Fig.9). The phenotype confirmed the protection afforded by CCR2+ cell depletion, as indicated by ERG results (Fig. 4) and OCT structural data (Fig. 5). However, it is important to note that the dysregulation of complements in microglia is not the sole mechanism for promoting photoreceptor cell death in rd10 mice. Photoreceptor degeneration also results from a combination of factors, including metabolic disorders, inflammatory factors, and ROS production, but the abnormal activation of the complement pathway is a critical factor.

The functions of C3 in neuronal cell degeneration remain controversial (Natoli et al., 2017; Silverman et al., 2019; King et al., 2023; Kulak et al., 2024; Garton et al., 2025). Current literature indicates that intracellular and extracellular C3 may play distinct functions (King et al., 2023; West et al., 2023). Intracellular C3 may play an additional role by acting in non-canonical ways via interactions with a distinct set of intracellular ligands, thereby maintaining autophagy homeostasis in the cell. In C3 knockout mice, the complete absence of C3 results in cell defects and even death under stress (King et al., 2019, 2023; West et al., 2023). In contrast, excessive C3 production can lead to the overactivation of the immune system. Both can disrupt immune balance and exacerbate neuron cell death. To protect cellular function and maintain homeostasis, it is necessary to maintain the normal physiological level of C3.

Various retinal pathologies can exhibit distinct phenotypes. We found that one-time or transient injuries, such as NaIO3-induced RPE injury and light damage, result in infiltrated MDMs that coexist with local microglia and form a new “homeostasis” with them at a later, stable stage in the retina. However, in persistent cell death/injury, such as in rd10 mice, the infiltrated MDMs remain transiently and are subsequently removed by local, activated microglia. Peripheral macrophage infiltration and survival require an empty niche and local support factors, such as CSF1 and/or IL34. Over-threshold transient injuries activated microglia, which migrated to the injury area, creating an empty niche that was occupied by infiltrating macrophages. The space's environment returned to its normal state after the injury stopped, providing the normal CSF1 and/or IL34 to support the differentiation of infiltrated MDMs into microglia-like cells and their coexistence. However, in persistent retinal degeneration, such as in rd10 mice, the local microglia and even infiltrated MDMs are continuously activated by injury, such as photoreceptor cell death, leading them to treat the MDMs as alien cells or dangerous pathogens and to execute them through phagocytosis. It could be the reason we can’t find a large number of MDMs in the later stages of the rd10 mouse retina, and why the gene expression pattern differs from that recently published (Paladini et al., 2025).

In summary, CCR2+ monocyte infiltration in the rd10 mouse retina promotes local inflammation, activated interferon response, and ROS production. More importantly, it activates the alternative complement pathway by increasing expression of C3 and CFB, while inhibiting CFH expression. This leads to increased iC3b/C3b deposition on the photoreceptor, accelerating cell death (Fig. 9). Therefore, blocking monocyte infiltration could be a therapeutic strategy to enable effective neuroprotection in retinal degenerative diseases.

## Methods and materials

### Experimental Animals

CCR2-CreER knock-in mice were generated by replacing CCR2 coding sequence with those coding for CreERt2. These mice were crossed into “floxed” transgenic lines coding for the marker tdTomato (tdT, JAX lab, #007914), and diphtheria toxin subtype A (DTA, JAX Lab, #009669), and into the rd10 (JAX lab, #004297) background to generate the following lines: (1) CCR2^CreER/+^;tdT^F/+^ (CCR2-tdT); (2) rd10;CCR2^CreER/+^;tdT^F/+^ (rd10-CCR2) and (3) rd10;CCR2^CreER/+^;tdT^F/+^;DTA^F/+^ (rd10-CCR2-DTA). Tamoxifen (Sigma, T5648) administration was used to induce tdT and DTA expression. The CCR2^CreER/+^;tdT^F/+^;CX3CR1^EGFP/+^(CCR2^CreER^-CX3CR1) line was generated by crossing CX3CR1-EGFP (JAX Lab, #005582) and CCR2^CreER/CreER^;tdT^F/F^ mice. CCR2-RFP (JAX lab, #017586) knock-in mice also crossed with CX3CR1-EGFP mice to generate CCR2-CX3CR1 mice. All animals were bred and housed in a National Institutes of Health animal facility under a 12-h light/dark cycle with food ad libitum. Experiments were conducted consistent with protocols (NEI606, NEI698) approved by the National Eye Institute Animal Care and Use Committee. They adhered to the ARVO Statement for the Use of Animals in Ophthalmic and Vision Research. All mice were confirmed to be free of RD8 contamination.

### tdTomato (tdT) induction and CCR2 expression cell ablation

To induce ER-mediated Cre recombination and expression of tdTomato in CCR2-expressing cells, two-month-old CCR2^CreER/+^;tdT^F/+^ mice were administered tamoxifen dissolved in corn oil (Sigma-Aldrich; 500 mg/kg dose of a 20 mg/ml solution) via oral gavage (two doses, one day apart). These animals were kept under standard vivarium conditions and used in subsequent experiments 1 week later. For both of tdtomato induction and CCR2 expression cell ablation, the tamoxifen dissolved in corn oil was given into rd10;CCR2^CreER/+^;tdT^F/+^ (rd10-CCR2) and rd10;CCR2^CreER/+^;tdT^F/+^;DTA^F/+^ (rd10-CCR2-DTA) mice continuously 3 times at P14, P15, and P16 (60–100ug/g body weight) through intraperitoneal injection (IP). For NaIO3-induced RPE injury mice, 100mg/kg body weight of tamoxifen was given to the CCR2^CreER/+^;tdT^F/+^ and CCR2^CreER/+^;tdT^F/+^;DTA^F/+^ mice through intraperitoneal injection.

### Blood and bone marrow granular cell analysis

Blood was collected from the right atrium. Briefly, two-month-old CCR2^CreER/+^;tdT^F/+^, CCR2^CreER/+^;tdT^F/+^;DTA^F/+^ or CCR2^CreER/+^;tdT^F/+^;CX3CR1^EGFP/+^(CCR2-CX3CR1) mice were anesthetized with an intraperitoneal injection of ketamine (90 mg/kg) and xylazine (8 mg/kg). After opening the chest, the heart was exposed. An EDTA-treated 22-gauge needle with a 3 mL syringe was inserted into the right atrium to collect all blood, which was then immediately transferred to 10 mL ACK Lysing buffer for incubation at room temperature for 5–10 minutes. Collect the white blood cells by centrifugation at 300 × g for 5 minutes at room temperature. Aspirate the supernatant, leaving approximately 50 μL to avoid disturbing the pellet, then add 5 mL of cold phosphate-buffered saline. Mix and centrifugate at 300 g for 5 minutes at 2–8°C. Aspirate the supernatant and resuspend the cells in 100 μL of PBS. The bone marrow cells were collected from the hind leg tibia, and both femur ends were cut with a scalpel or sharp scissors to clean both ends. Flushing the bone marrow cells out with a 10 mL syringe and 25-gauge needle with 10 mL HBSS, then collecting the cells and centrifuging at 300g for 10 minutes at 4 °C. Washing 1 time with PBS. The blood and bone marrow granular cells were stained with CD11b-488 (eBioscience, # 53-0112-82), Ly6C (ThermoFisher Scientific, RM3021/50-5931-82), and CD45 (eBioscience, 30-F11) antibodies (1:30), and incubated on the ice for 20 minutes. After 2^nd^ antibody staining, the cells were fixed with 2% PFA for 20m and washed 2 times with PBS. The cell pellet was resuspended in 10 μL of PBS, transferred to a gelatin-coated slide, smeared onto the slide, and dried at room temperature. After covering them with a mounting medium containing DAPI (Vector Labs) and a coverslip, images were captured using an Olympus FV3000 and a Nikon Eclipse Ti2 confocal microscope.

### RPE cell injury by NaIO_3_ administration

In the NaIO3-induced RPE injury model, two-month-old CX3CR1^GFP/+^;CCR2^RFP/+^ mice, CX3CR1^GFP/+^;CCR2^CreER/+^;tdT^F/+^ mice, CCR2^CreER/+^;tdT^F/+^ mice and CCR2^CreER/+^;tdT^F/+^;DTA^F/+^ mice (previously administered oral tamoxifen 1 week ago) were administered a single dose of NaIO3 (Honeywell Research Chemicals, 30 mg/kg body weight) via intraperitoneal injection. Animals were euthanized, and their retinas were analyzed during 1 to 180 days following NaIO3 administration.

### Light damage models

Experimental animals were dark-adapted in a dark room for 7 days and then subjected to pupillary dilation with topical tropicamide (1%; Alcon) and phenylephrine (10%; Alcon). After full dilation, the animals were exposed to 2 × 10^4^ lux of diffuse white fluorescent light (Sunlite Manufacturing) for 2 hours. After light exposure, the mice were maintained in typical ambient cyclic light conditions (100 lux, 12:12 h), where they were housed. The mice were harvested 3 and 7 days after light damage.

### Electroretinography (ERG) detection and analysis

ERG was recorded using an Espion E3 system (Diagnosys). Mice were anesthetized with an intraperitoneal injection of ketamine (90 mg/kg) and xylazine (8 mg/kg) after dark adaptation overnight. Pupils were dilated with topically administered tropicamide (1%, Alcon) and phenylephrine (2.5%, Alcon), and proparacaine hydrochloride (0.5%, Alcon) was used for topical anesthesia. Flash ERG recordings were obtained simultaneously from both eyes with gold wire loop electrodes, with the reference electrode placed in the mouth and the ground subdermal electrode at the tail. ERG responses were obtained at increasing light intensities over the ranges of 1 × 10^−4^ to 10 cd·s/m^2^ under dark-adapted conditions and 0.3 to 100 cd·s/m^2^ under a rod-saturating background light. The stimulus interval between flashes varied from 5 s at the lowest stimulus to 60 s at the highest stimulus. 2 to 20 responses were averaged depending on flash intensity. ERG signals were recorded with 0.3-Hz low-frequency and 300-Hz high-frequency cutoffs sampled at 1 kHz. Analysis of a-wave and b-wave amplitudes was performed using Espion ERG Data Analyzer software (version 6.0.54). The a-wave amplitude was measured from the baseline to the negative peak, and the b-wave was measured from the a-wave trough to the maximum positive peak. Statistical comparisons of ERG amplitudes between animals of different genotypes were analyzed using a two-way ANOVA.

### In vivo OCT imaging of the mouse retina

Mice were anesthetized with an intraperitoneal injection of ketamine (90 mg/kg) and xylazine (8 mg/kg), and their pupils were dilated with 1% tropicamide and 2.5% phenylephrine (Alcon). Retinal structure was assessed using an OCT imaging system (Bioptigen; InVivoVue Software). Volume scans comprised 100 horizontal sequential B-scans (composed of 1,000 A-scans each), spanning an en-face retinal area of 1.4 by 1.4 mm centered on the optic nerve head. Retinal thickness measurements were performed in the superior and inferior retinal quadrants at a radial distance of 0.6 mm from the optic nerve head using the manufacturer’s measurement scale (Bioptigen; Diver) and averaged for each eye. The thickness of the total retinal layer (ERL), defined as the distance from the nerve fiber layer to the RPE basal, and outer retinal layer (ONL), defined as the distance from the OPL (including OPL) to the basal of the RPE, was measured from OCT images after manual retinal segmentation. The presence of shallow retinal detachments was manually evaluated in the entire scan field and excluded from measurement.

### Flow cytometry assay

The blood was collected from the right atrium, and the bone marrow cells were splashed from the tibia as described above (Blood and bone marrow granular cell analysis section). The mice include no tamoxifen induction, 1 day after tamoxifen induction of both CCR2^CreER/+^;tdT^F/+^ and CCR2^CreER/+^;tdT^F/+^;DTA^F/+^ mice. After digestion in ACK buffer (Gibco), the cells were stained with Ly6C-PerCP Cyanine5.5 and CD11b 488. 500ul of suspended cells were analyzed using a Beckman Coulter CytoFlex NUV (Brea, CA) flow cytometer.

### Immunohistochemistry

Fixed eyecups were prepared for the whole retina flat mount and cryosections. The retinal samples were treated with 1% Triton for 1 hr for the flat mount and with 0.25% Triton in PBST (0.25% Tween 20) for 0.5 hrs for the retinal section. After blocking, the following primary antibodies were used: Iba1 ( 1:500 Wako), Iba1 (SYSY, 311H9H4, 1:300), CD11b (BioRad, 1:100), Ki67 (eBioscience, # 50-5698-82, 1:40), TMEM119 (Synaptic System, 1:500), anti-P2RY12 (1:100, ThermoFisher, #PA5-77671 and Sigma, #HPA014518), anti-mouse CD68 (1:200, BioRad, #MCA1957), anti-mouse CD45 (1:100, Bio-Rad, #MCA1388), Ly6C (ThermoFisher Scientific, clone#RB6-8C5, 1:100), MHC2 (IA/IE, BD Bioscience, 1:30), cone Arestin (1:200, Millipore, #AB15282), Glutamate synthesis (GS, 1:200, Millipore), CD74 (1:100, clone number:VIC-Y1, Thermofisher), Lectin PNA From Arachis hypogaea (peanut, ThemoFisher), C3 (1:200, Hycult, HM1045), C3/iC3b/C3C (1:200, Hycult, HM1078), C3b/iC3b/C3C (1:100, Hycult, clone 2/11, HM1065). Secondary antibodies raised in goat or donkey and conjugated to either Alexa 488, Alexa 568 or Alexa 647 (1:300, ThermoFisher and Jax Lab) were used. The images were taken with the Olympus 1000/3000 and Nikon Eclipse Ti2 confocal microscope.

### Sholl analysis and mean IntDen measurement

Microglial morphology was analyzed using the Fiji Plugins program for Neuroanatomy. After the microglial image was processed with threshold and binary settings, it was skeletonized. From Plugins Neuroanatomy, set up: step size=10; End radius = 120; the center of the soma was set up using Wand tool. After running the Sholl analysis, the data table was saved. The total of 16–20 cells from 4 retinas in each group were analyzed for correlation coefficients and plotted in Prism. The protein level of gene expression was determined using the mean IntDen measurement with Fiji software after random cell selection.

### Density of PNA+ photoreceptor cell analysis

For cone photoreceptor cell density analysis, we stained cells with peanut agglutinin (PNA) and acquired images using confocal microscopy (Olympus 3000). The images were analyzed in ImageJ (FIJI) using the particle analysis function after threshold adjustment. Cell coverage percentage was used for analysis. To assess macrophage phagocytic ability, the mean intensity of CD68 staining was analyzed using ImageJ. A total of 4 images of each retina and a total of 3 biological replicates were used for analysis.

### Retinal microglia and MDM isolation

The retinal microglia isolation was described previously (Ma et al., 2009). Briefly, the P19 and P23 rd10-CCR2 and rd10-CCR2-DTA mice were euthanized, and the eyes were immediately enucleated and transferred into ice-cold Hank’s balanced salt solution (HBSS). The retinas were isolated by dissection and transferred into 0.2% papain solution and incubated at 8°C for 45 minutes, and then at 28°C for 7 minutes. The dissociated retinal cells were pelleted, neutralized, and washed once with albumin, glucose, and DNase I. The cellular pellet was resuspended in 100 uL of staining buffer (catalog no. 554656, BD Pharmingen, San Diego, CA, USA) containing a fluorescein isothiocyanate (FITC)-conjugated antibody to CD11b (1:50; catalog no.11–0112, eBioscience, San Diego, CA, USA), and incubated for 20 minutes on ice. The cells were washed twice in 10 mL of staining buffer containing 2 mM ethylenediaminetetraacetic acid (EDTA) and suspended in 0.5 mL of staining buffer plus EDTA and DAPI. Microglia and MDMs were isolated by fluorescence-activated cell sorting (FACS) (BD FACSAria II Flow Cytometer; BD, Franklin Lakes, NJ, USA) at the NHLBI Flow Cytometry Core Facility. FACS sorting set up as below: Resident microglia: CD11b+ and tdT−; MDMs: tdtomato+ with or without CD11b+. The microglia, MDMs and both CD11b & tdtomato negative cells were sorted into 2 mL tubes with 1 mL of TRIzol, respectively, and stored at −80 °C for further RNA isolation.

### Bulk RNA sequencing

The total RNA was isolated from sorted cells in Trizol (Invitrogen) following the manufacturer's instructions. Briefly, 0.2 mL of chloroform (Sigma) was added to 1ml of Trizol with the cells and incubated for 3 minutes at room temperature. Centrifuge the samples at 3500g for 40 minutes at 4 °C. Transfer 400–500 μL of the aqueous phase to a fresh tube and add an equal volume of 70% ice-cold ethanol to the tube. Then, following the RNA extraction kit (Qiagen, Qiagen RNeasy Plus Micro kit) for binding RNA and purification. The RNA quality and quantity were measured with a Bioanalyzer (Agilent, Agilent 2100). The RNAs with RIN numbers over 7 were used for RNAseq. The RNA-seq was performed at AmpSeq Biotechnology company (Gaithersburg, MD). The cDNA library was prepared using the VAHTS Universal V10 RNA-seq Library Prep Kit for Illumina (Vazyme). The quality was assessed using Qubit (ThermoFisher) and qPCR, and the fragment size was analyzed with Qsep. The sequencing was run on Element Biosciences AVITI (Element Biosciences) using a 2X150 cycle sequencing kit (Illumina). The RNASeq quantification pipeline begins by using fastp (fastp, v0.23.1) to trim adapters and low-quality bases from the raw sequencing reads. Next, the trimmed reads are aligned to the mouse reference genome (mm39.fa) using the STAR (v2.7.11) aligner, which efficiently maps the reads to their corresponding genomic locations. Following alignment, the Salmon package (v1.10.2) is employed to perform quantification at the transcript level. Salmon employs a lightweight alignment-based approach to estimate transcript abundances, considering both the mapped reads and the known transcript sequences. Finally, the tximport package (v1.30.0) in R is utilized to import the transcript-level quantification results generated by Salmon and summarize them at the gene level. This process involves mapping the transcripts to their corresponding genes and aggregating the abundance estimates, resulting in gene-level quantification data that can be used for downstream analysis and interpretation.

Following the trimming step, quality control is performed on the trimmed sequences using FastQC (v0.11.9). To summarize the FastQC results across all samples, MultiQC (v1.11) is employed. MultiQC aggregates the individual FastQC reports and generates a single, interactive report that allows for easy comparison and visualization of the QC metrics across the entire dataset. The gene-level quantification data includes two main types of quantification measures: raw count data and TPM (Transcripts Per Million) data. Raw count data represents the number of reads that map to each gene, providing a direct measure of gene expression. TPM data, on the other hand, is a normalized measure of gene expression that takes into account the length of each gene and the total number of mapped reads in each sample. TPM values are calculated by dividing the read counts by the length of each gene in kilobases, and then normalizing the resulting values to account for differences in library size across samples. TPM data allows for more accurate comparison of gene expression levels between samples and across different genes. Both raw count data and TPM data can be used for downstream analysis, such as differential expression analysis (DESeq2).

### Statistical Analysis

All data were analyzed using GraphPad Prism (Version 10). The results are expressed as mean ± SEM for experiments conducted at least in triplicate. An unpaired two-tailed Student’s t-test was used to assess differences between the two groups; non-Gaussian distributions were evaluated with a Mann-Whitney test. In the Sholl analysis of microglial and MDM morphology, the correlation coefficient was used to demonstrate the linear association. For all ERG data, two-way ANOVA was used to test for significance. A value of P < 0.05 was considered statistically significant.

## Supplementary Material

Supplement 1

Supplementary Files

This is a list of supplementary files associated with this preprint. Click to download.

• Suppl.Table1ResultsofgenesetsenrichmentanalysisofretinalmicrogliainP23rd10micenodepletionvs.depletion.xlsx

• Suppl.Table2ResultsofgenesetsenrichmentanalysisintheretinasofP23rd10miceMDMvs.Microglia.xlsx

## Data Availability

All RNA-seq data are available in NCBI, project ID: PRJNA1368926.
