# Supplementary material for: Infiltrating monocytes augment alternative complement activation and exacerbate inherited retinal degeneration in a mouse model
Source: Res Sq. 2026 May 28:rs.3.rs-8734757. Preprint. [Version 1] doi: 10.21203/rs.3.rs-8734757/v1 (PMC13317638; doi:10.21203/rs.3.rs-8734757/v1)
Supplement: Supplement 1 [file NIHPPRS8734757V1-supplement-1.pdf]

Supplement figures and figure legends

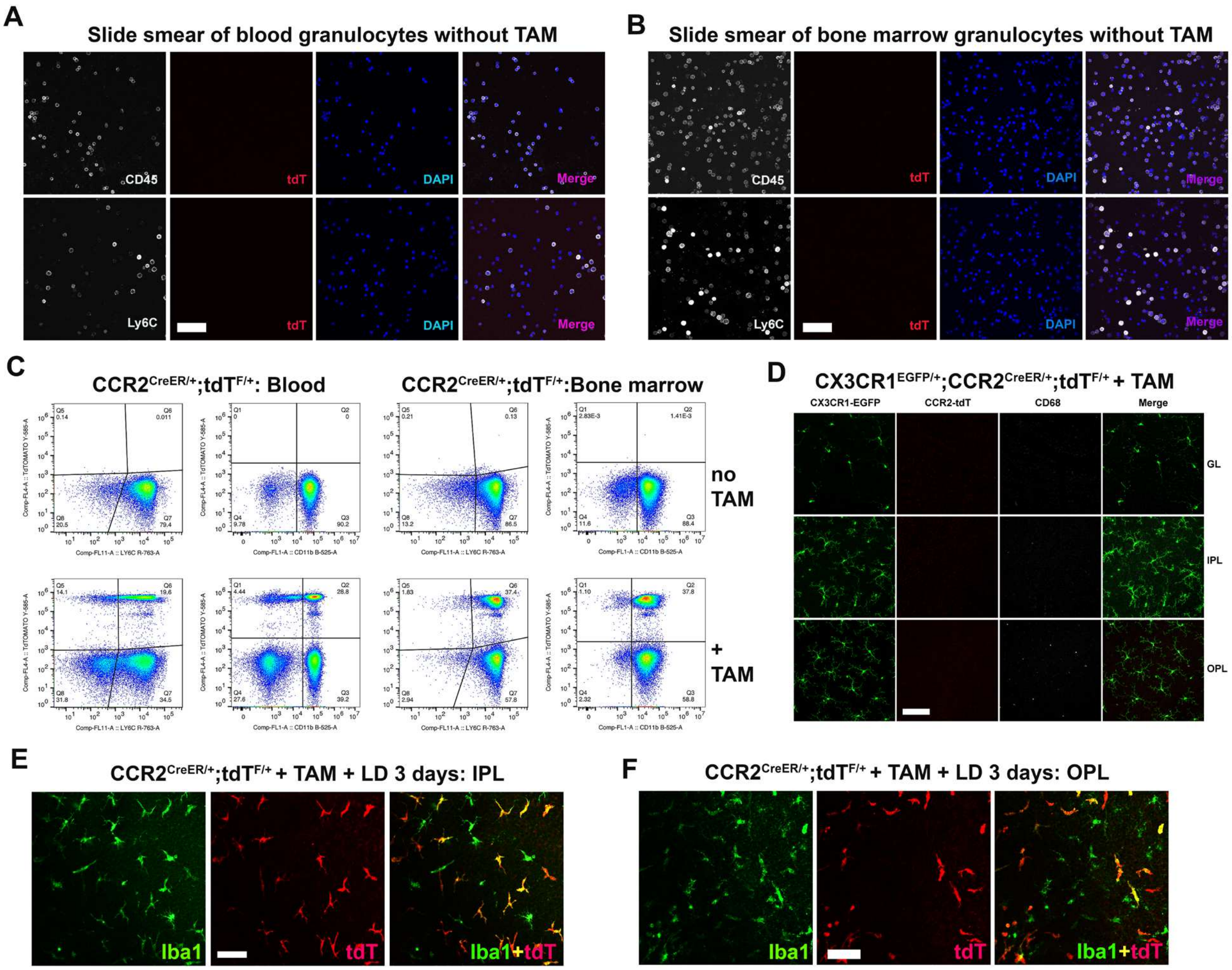

Fig. 1. Suppl. CCR2$^{CreER/+}$;tdT$^{F/+}$ mice are reliable for tracking peripheral monocyte-derived macrophages (MDM). CreER recombinase is inducible and shows no leakage, while tdTomato efficiently labels most myeloid cells in the blood and bone marrow. No tdtomato+ cells found in the blood (A) and bone marrow (B) without tamoxifen induction in CCR2$^{CreER/+}$;tdT$^{F/+}$ mice. Scale bar = 60 µm. (C) Flow cytometry showed no tdTomato expression in Ly6C+ and CD11b+ cells without tamoxifen induction (upper panels). Following 1 day of tamoxifen administration, robust tdTomato expression was observed in these myeloid cell populations (lower panels). (D) No CCR2-tdT+ cell infiltration in normal 2-month-old CCR2$^{CreER/+}$;tdT$^{F/+}$ mice after tamoxifen administration. The microglial cells in the normal mouse retina exhibit very mild CD68 staining. Scale bar = 60 µm. (E) and (F) Retinal light damage (LD) caused CCR2-tdT+ cell infiltration in CCR2$^{CreER/+}$;tdT$^{F/+}$ mice after tamoxifen administration. Light damage induced CCR2-tdT+ cell infiltration to retinal IPL (E) and OPL (F) (3 days after light damage). Scale bar = 60 µm.

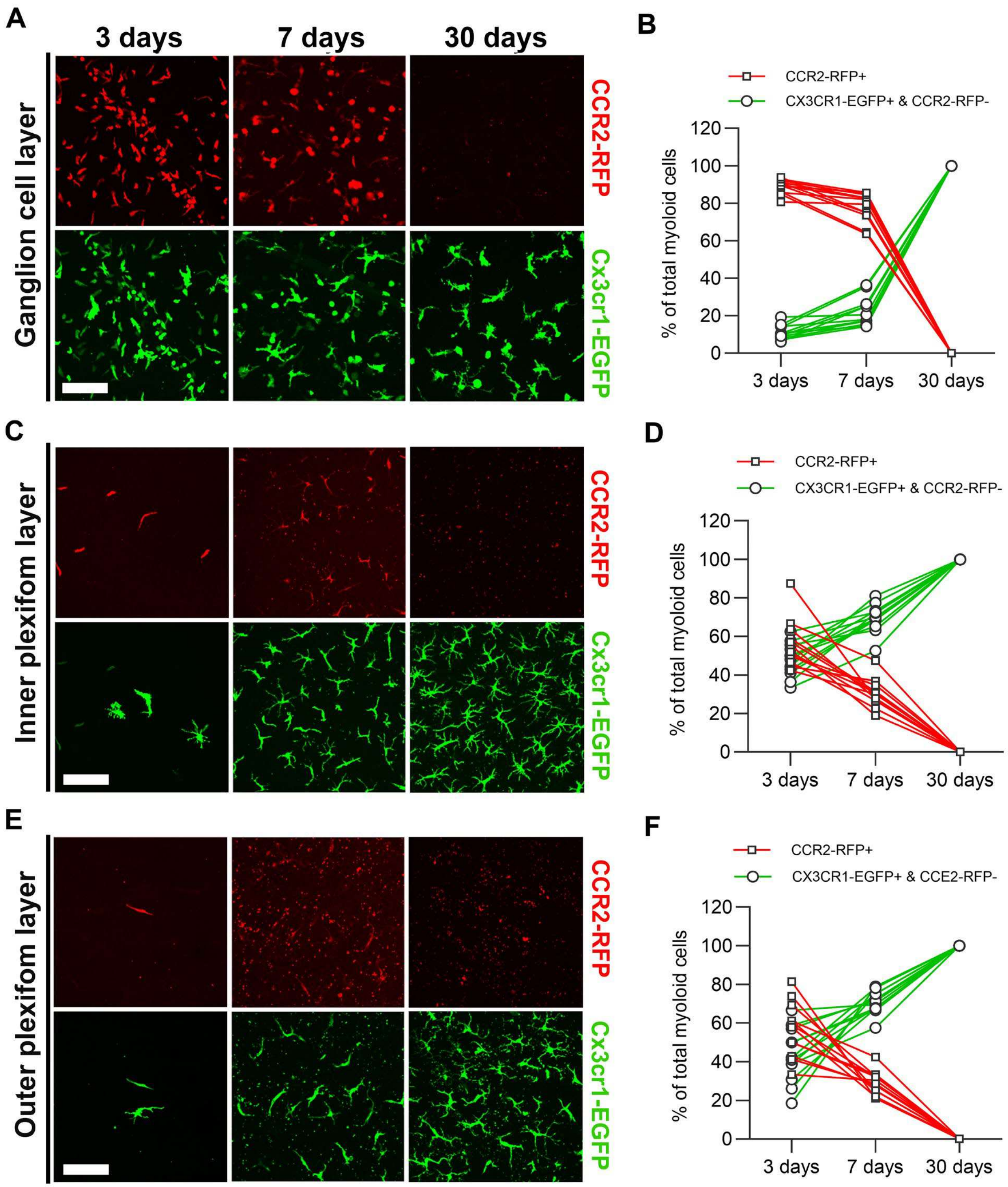

Fig. 2 suppl. CCR2-RFP knock-in mice only track peripheral macrophages for a short time. In CCR2$^{RFP/+}$;CX3CR1$^{EGFP/+}$ mice, a NaIO3-induced RPE injury model was generated. CCR2-RFP+ and CX3CR1-EGFP+ cells were shown in the retinal GL (A, B), IPL (C, D), and OPL (E, D), RFP+ cells were only shown in 3 and 7 days, but disappeared 30 days after RPE injury. However, CX3CR1-EGFP+ cells were shown at all times. Scale bar = 60 μm.

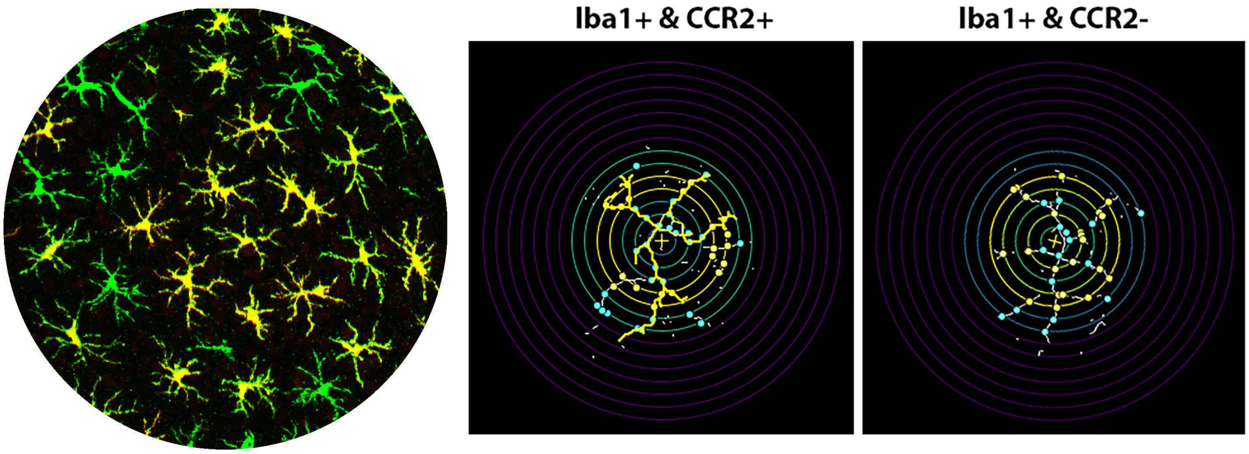

Fig. 3. Suppl. 1. Representative images of cell morphology analysis of infiltrated CCR2+ & Iba1+ macrophages and CCR2- & Iba1+ resident microglia using Sholl analysis. The image shows the OPL of a mouse retinal flatmount after 40 days of NaIO3 administration.

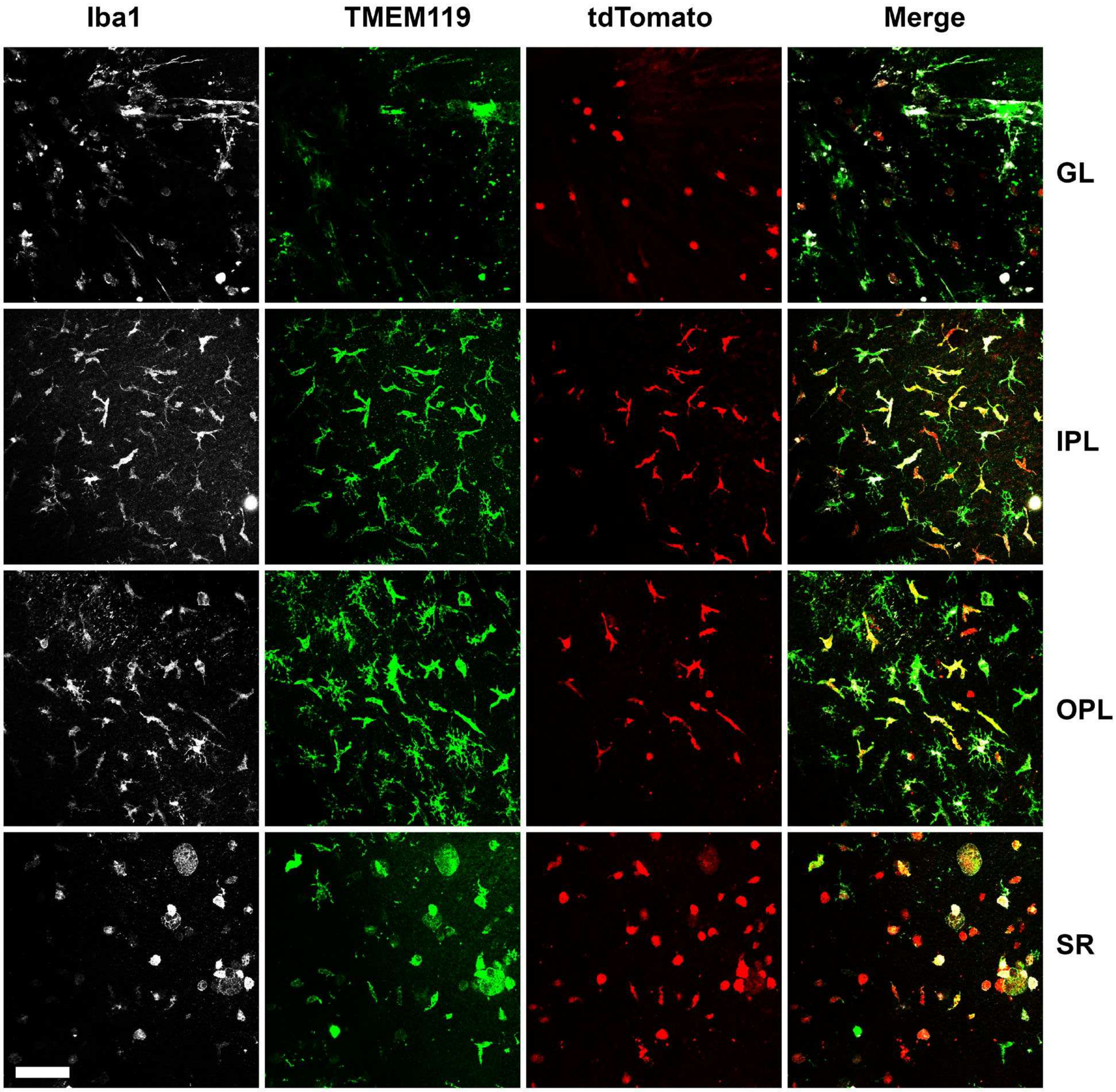

Fig. 3. Suppl. 2. Infiltrated CCR2+ macrophages expressed TMEM119 in the light-damage 3 days in $CCR2^{CreER/+}$;$tdT^{F/+}$ mouse retinas after tamoxifen induction. Retinal flat mount images showed both infiltrated CCR2-tdT+ macrophages and tdT- resident microglia express TMEM119 in GL, IPL, OPL and subretinal space (SR). Scale bar 60 µm.

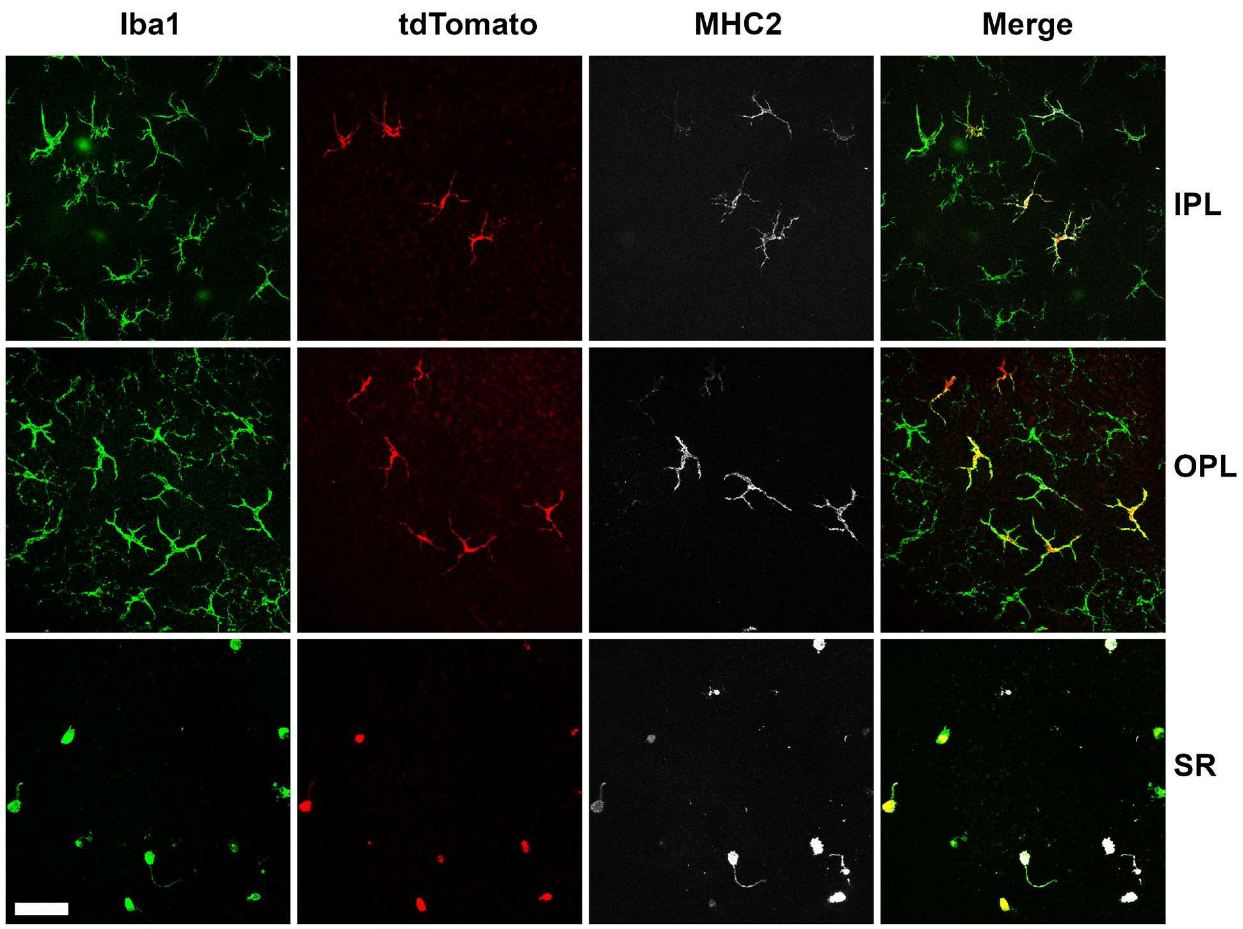

Fig. 3. Suppl. 3. Both infiltrated CCR2+ macrophages and CCR2- resident microglia expressed MHC2 in the light-damaged 7-day in $CCR2^{CreER/+}$;$tdT^{F/+}$ mouse retinas. Scale bar = 60 µm.

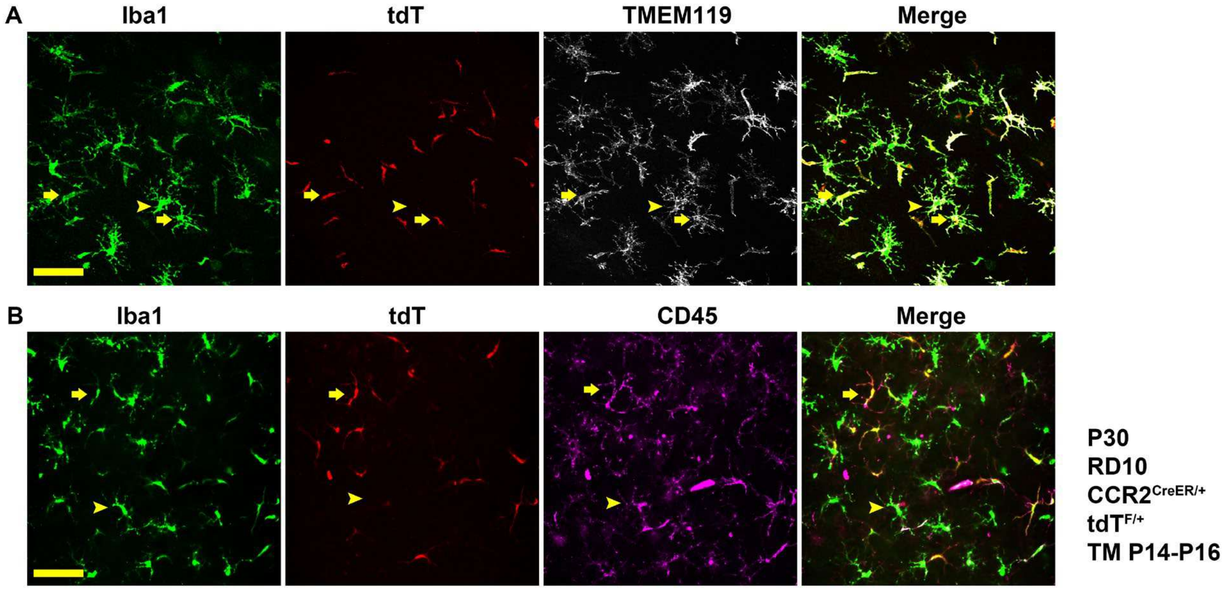

Fig. 3. Suppl. 4. In P30 rd10;CCR2$^{CreER/+}$;tdT$^{F/+}$ mice retina after tamoxifen induction from P14 to P16, both the infiltrated CCR2+ MDMs (arrow) and local Iba1+ and CCR2- microglia (arrow head) express TMEM119 (A) and CD45 (B). Scale bar = 60um.

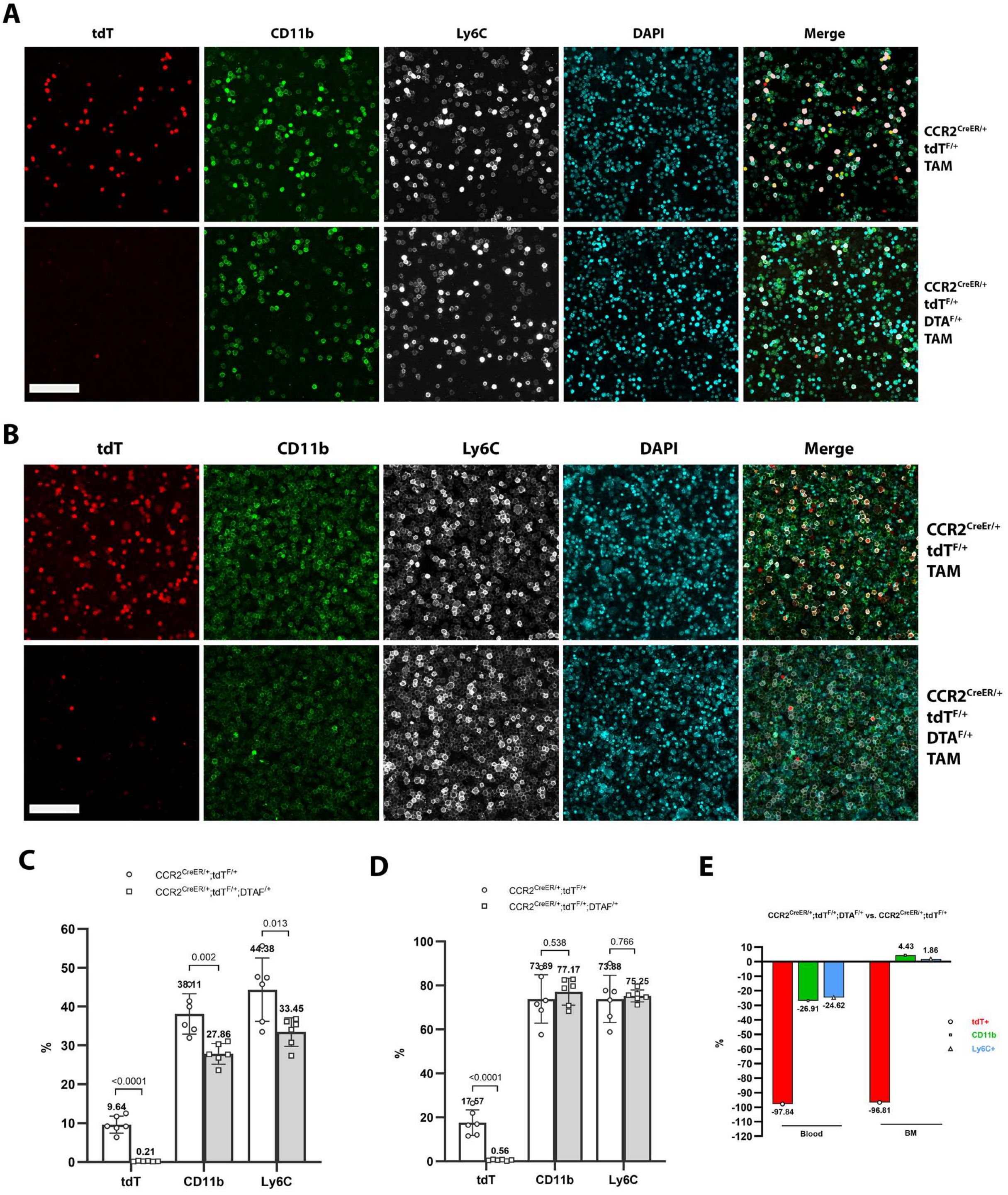

Fig. 4. Suppl. 1. The blood and bone marrow granulocytes staining immediately after continued 3 days of tamoxifen IP injection in CCR2$^{CreER/+}$;tdT$^{F/+}$ and CCR2$^{CreER/+}$;tdT$^{F/+}$;DTA$^{F/+}$ mice. A. Blood granulocytes

staining with CD11b (green), Ly6C (white) and DAPI (cyan), the tdT+ cells decreased significantly in CCR2$^{CreER/+}$;tdT$^{F/+}$;DTA$^{F/+}$ (lower panel) mice. Scale bar = 60 µm. B. Bone marrow granulocytes staining with CD11b (green), Ly6C (white) and DAPI (cyan), the tdT+ cells decreased significantly in CCR2$^{CreER/+}$;tdT$^{F/+}$;DTA$^{F/+}$ (lower panel) mice. Scale bar = 60 µm. C. The percentage of tdT+, Ly6C+, and CD11b+ cells in all blood granulocytes (DAPI+). D. The percentage of tdT+, Ly6C+, and CD11b+ cells in all bone marrow granulocytes (DAPI+). E. The percentage changes of tdT+, CD11b+ and Ly6C+ cells in blood and bone marrow were compared between CCR2$^{CreER/+}$;tdT$^{F/+}$;DTA$^{F/+}$ versus CCR2$^{CreER/+}$;tdT$^{F/+}$ mice after tamoxifen induction.

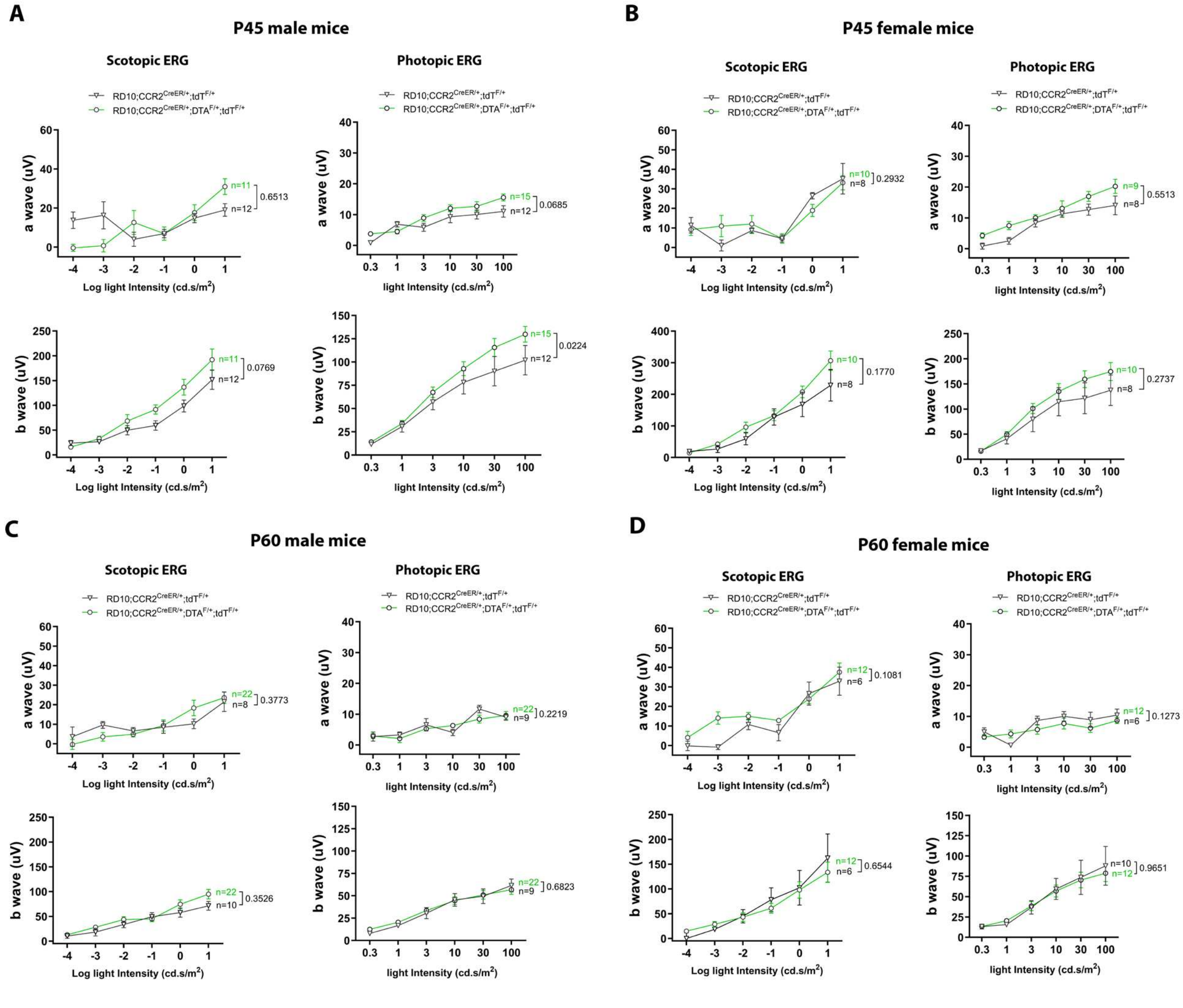

Fig. 4. Suppl. 2. Ablation of peripheral monocytes did not protect retinal function in the later stage of retinal degeneration in rd10 mice. A. The ERG results from P45 male RD10 mice. B. The ERG results from P45 female rd10 mice. C. The ERG results from P60 male rd10 mice. D. The ERG results from P60 female rd10 mice. All data were analyzed using a two-way ANOVA.

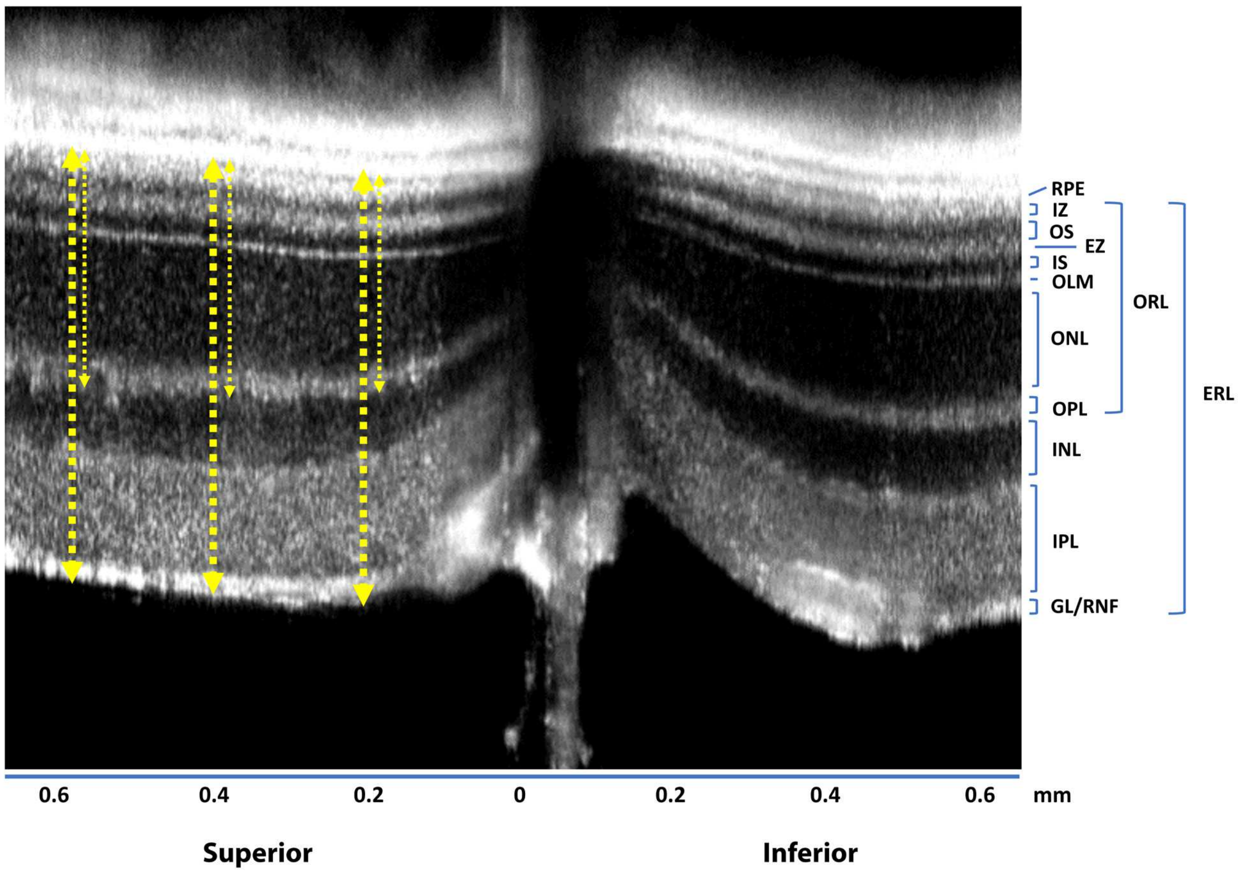

Fig. 5. Suppl. 1. Diagram shows the thickness measurement of the retina and the retinal layers. The measurement size is 0.6mm away from the optic nerve head (ONH) at the superior and inferior regions of the retina. Outer retinal layer (ORL): measure the thickness from the basal of RPE to the edge of the OPL, and entire retinal layer (ERL): measure the thickness from the basal of the RPE to GL/RNF. IZ: Interdigitation Zone, EZ: ellipsoid zone, OS: outer segment, IS: inner segment, OLM: outer limiting membrane, ONL: outer nuclear layer, OPL: outer plexiform layer, INL: inner nuclear layer, IPL: inner plexiform layer, GL/RNF: ganglion cell layer/retinal nerve fiber layer.

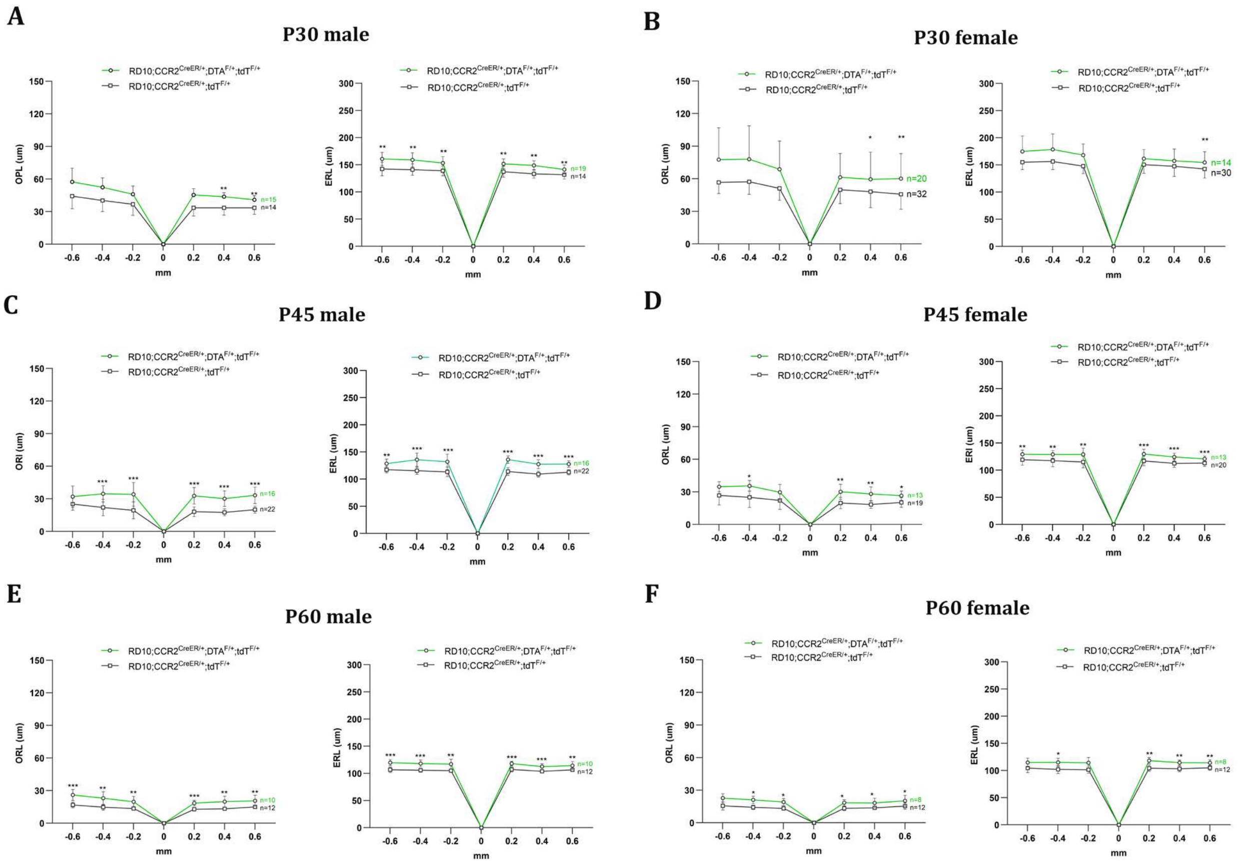

Fig. 5. Suppl. 2. Depletion of peripheral monocytes preserves the retinal thickness in the retinal degeneration of rd10 mice. (A) The thickness of the outer retinal layer (ORL, Left) and the entire retinal layer (ERL, Right) in P30 male rd10;CCR2$^{CreER/+}$;tdT$^{F/+}$ and rd10;CCR2$^{CreER/+}$;tdT$^{F/+}$;DTA$^{F/+}$ mice with tamoxifen (TAM) administration. (B) The thickness of the ORL (Left) and the ERL (Right) in P30 female retinas. (C) The thickness of the ORL (Left) and the ERL (Right) in P45 male retinas. (D) The thickness of the ORL (Left) and the ERL (Right) in P45 female retinas. (E) The thickness of the ORL (Left) and the ERL (Right) in P60 male retinas. (F) The thickness of the ORL (Left) and the ERL (Right) in P60 female retinas.

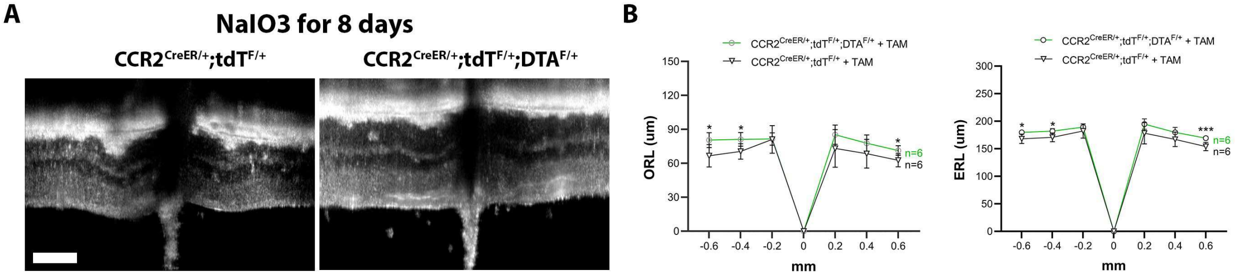

Fig. 5. Suppl. 3. Depletion of peripheral monocytes preserves the retinal thickness at 8 days of RPE injury induced by NaIO3. (A) The OCT images of CCR2$^{CreER/+}$;tdT$^{F/+}$ (left) and CCR2$^{CreER/+}$;tdT$^{F/+}$;DTA$^{F/+}$ mice retina. Scale bar = 0.1mm. (B) The thickness of ORL (left) and ERL (right) comparison between CCR2$^{CreER/+}$;tdT$^{F/+}$ and CCR2$^{CreER/+}$;tdT$^{F/+}$;DTA$^{F/+}$ mice retinas.

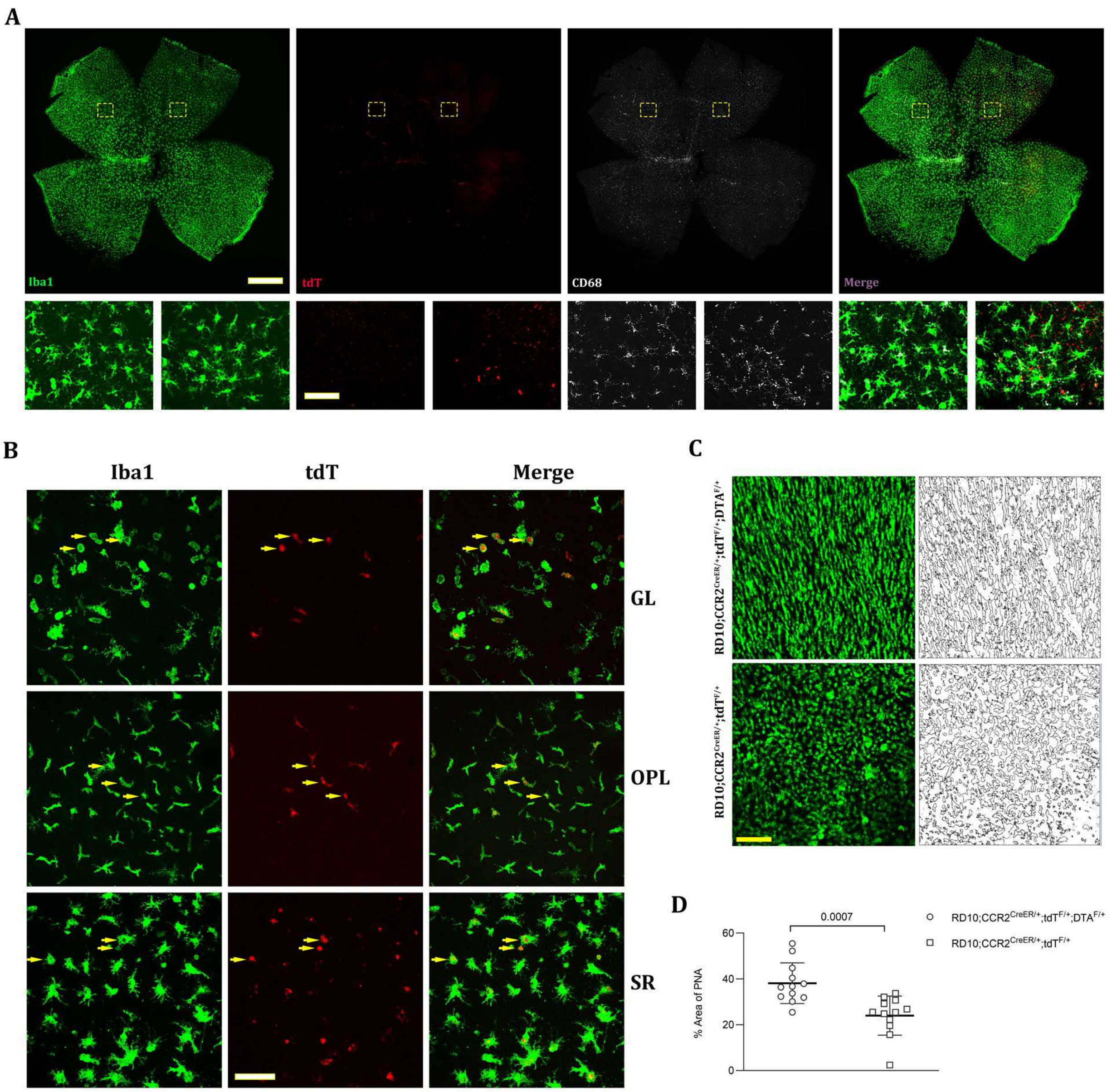

Fig. 6. Suppl. Ablation of peripheral CCR2+ monocytes decreases the microglia cell clustering and the ability of phagocytosis. A. Depletion of peripheral monocytes decreased the microglia clustering (whole mount, p17) in rd10;CCR2$^{CreER/+}$;tdT$^{F/+}$;DTA$^{F/+}$ mice retina. Scale bar = 1 mm. The magnified area showed a more even distribution of microglia. Scale bar = 125 µm. B. The representative images showed resident microglia phagocytosing infiltrated CCR2+ monocytes (P24) as indicated by the arrow; scale bar = 200 µm. C. The representative images demonstrated how the PNA area was analyzed using ImageJ. Scale bar = 60 µm. D. The percentage of the area of PNA occupied.

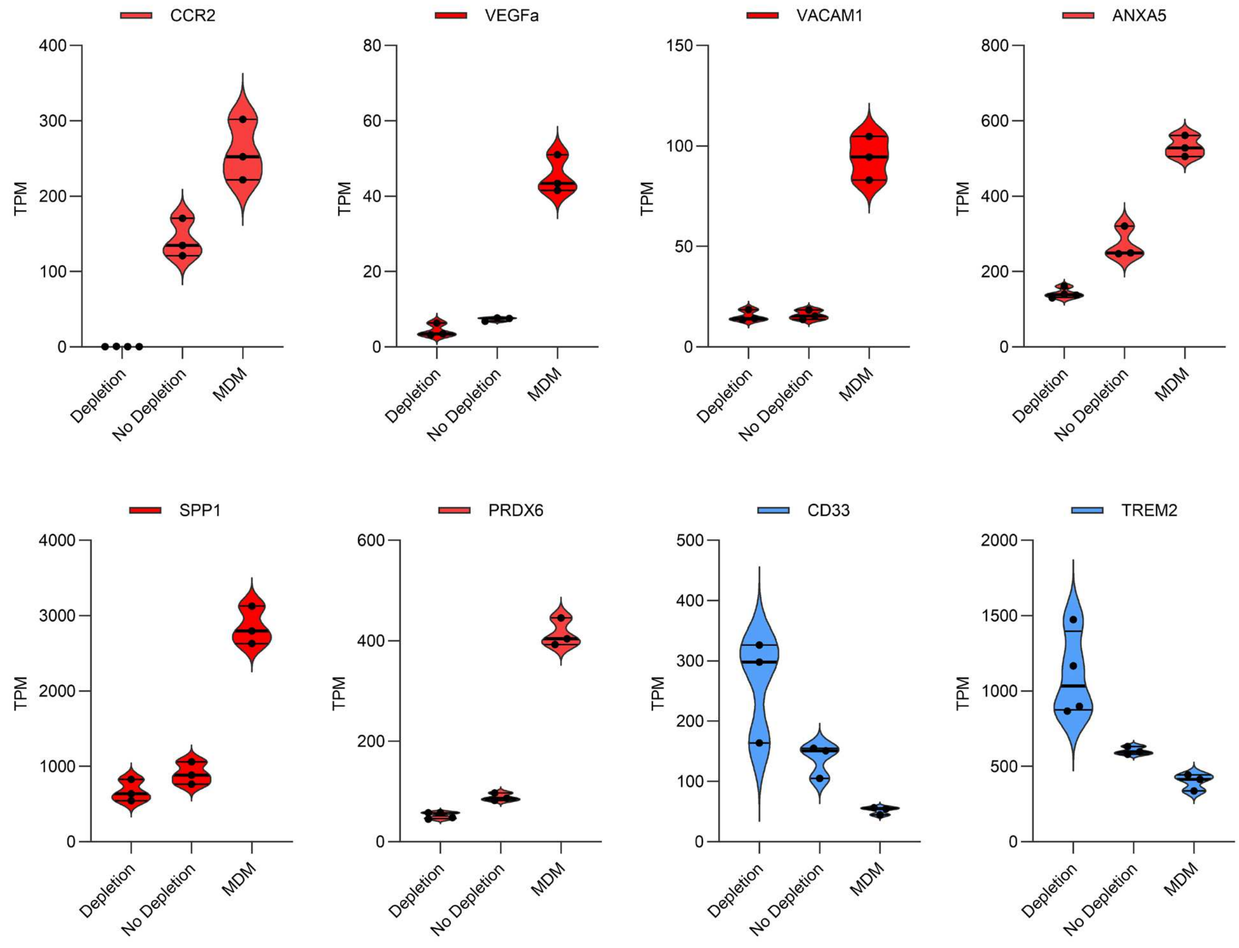

Fig. 7. Suppl. 1. Some representative genes involved in the gene sets enrichment in Fig. 7D.

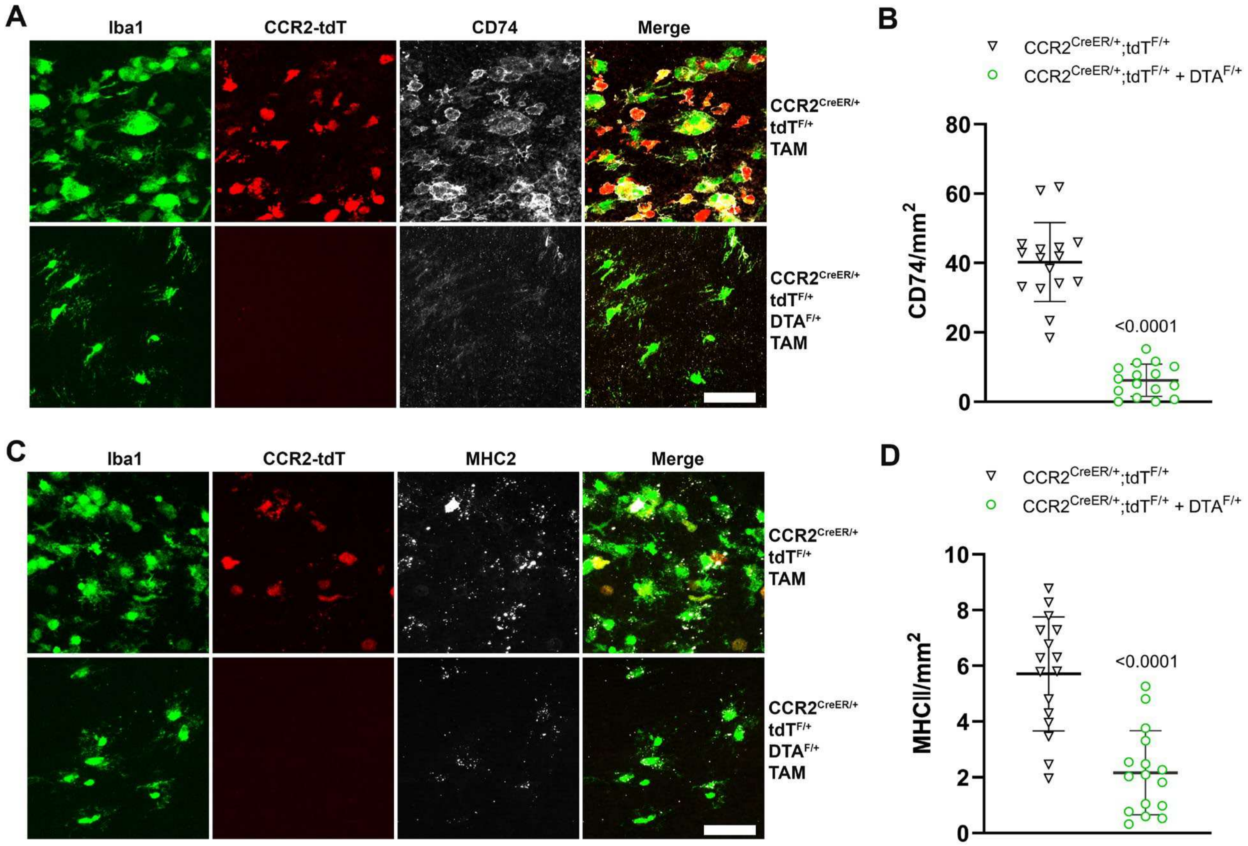

Fig. 7. Suppl. 2. Ablation of peripheral monocytes decreased CD74 and MHC2 production in microglia cells. Immunocytochemistry staining of Iba1, CD74 (A) and MHC2 (C) in the subretinal space of P19 rd10;CCR2$^{CreER/+}$;tdT$^{F/+}$ and rd10;CCR2$^{CreER/+}$;tdT$^{F/+}$;DTA$^{F/+}$ mice retina showed that CD74 (A, B) and MHC2 (C, D) were highly produced in microglia in the CCR2+ cell infiltration retina (upper panel) but decreased production in the CCR2+ cell depletion mouse retina (lower panel). Scale bar = 60 μm.

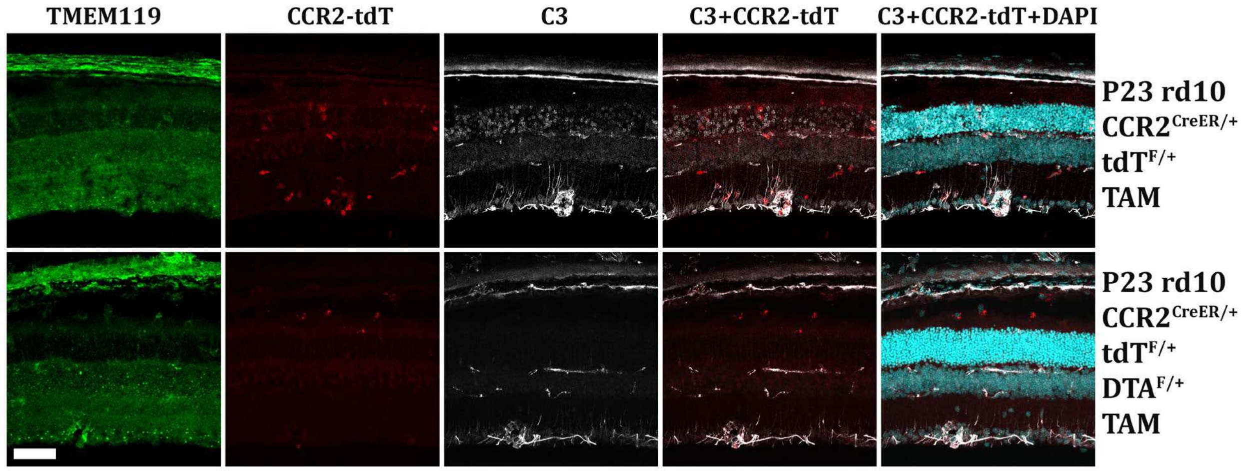

Fig. 8. Suppl. 1. Ablation of peripheral monocytes decreased C3 production. Immunohistochemistry staining of C3 on P23 rd10 mouse retinas showed that it was highly produced in Müller cells and deposited on photoreceptor cells in CCR2+ cell infiltration retina (upper panel), but decreased its

production in Müller cells and was absent from deposition in photoreceptors in CCR2+ cell depletion mouse retina (lower panel). Scale bar = 40 µm.

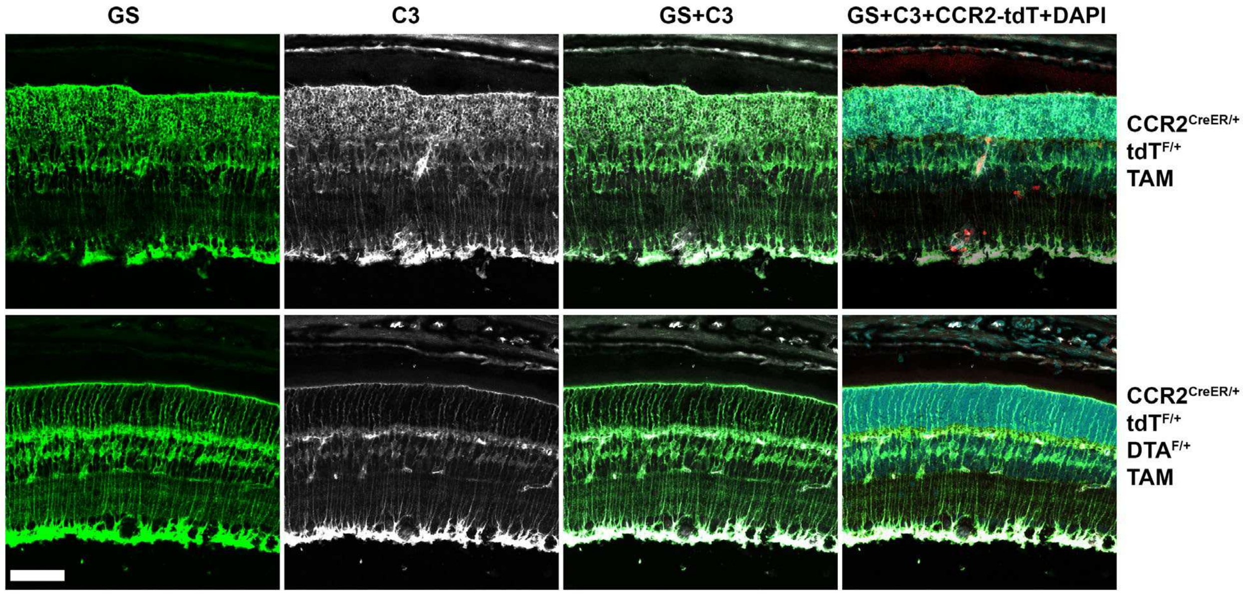

Fig. 8. Suppl. 2. Ablation of peripheral monocytes decreased C3 production in Müller cells. Immunohistochemistry staining of Glutamate synthesis (GS) and C3 on P23 rd10 mouse retinas showed that C3 was highly produced in Müller cells in the CCR2+ cell infiltration retina (upper panel), but decreased production in the CCR2+ cell depletion mouse retina (lower panel). Scale bar = 40 µm.
